# FOXK1 regulates malignant progression and radiosensitivity through direct transcriptional activation of CDC25A and CDK4 in esophageal squamous cell carcinoma

**DOI:** 10.1038/s41598-023-34979-y

**Published:** 2023-05-12

**Authors:** Xiaoxu Li, Juntao Lu, Lei Liu, Fei Li, Tongxin Xu, Liying Chen, Zhaoyang Yan, Yan Li, Wei Guo

**Affiliations:** 1 Laboratory of Pathology, Hebei Cancer Institute, The Fourth Hospital of Hebei Medical University, Jiankang Road 12, Shijiazhuang, 050011 Hebei China; 2 Department of Radiation Oncology, The Fourth Hospital of Hebei Medical University, Shijiazhuang, Hebei China; 3 Department of Thoracic Surgery, The Fourth Hospital of Hebei Medical University, Shijiazhuang, Hebei China

**Keywords:** Oesophageal cancer, Oncogenes, Radiotherapy

## Abstract

Esophageal squamous cell carcinoma (ESCC) is a serious malignancy with poor prognosis, necessitating identification of oncogenic mechanisms for novel therapeutic strategies. Recent studies have highlighted the significance of the transcription factor forkhead box K1 (FOXK1) in diverse biological processes and carcinogenesis of multiple malignancies, including ESCC. However, the molecular pathways underlying FOXK1’s role in ESCC progression are not fully understood, and its potential role in radiosensitivity remains unclear. Here, we aimed to elucidate the function of FOXK1 in ESCC and explore the underlying mechanisms. Elevated FOXK1 expression levels were found in ESCC cells and tissues, positively correlated with TNM stage, invasion depth, and lymph node metastasis. FOXK1 markedly enhanced the proliferative, migratory and invasive capacities of ESCC cells. Furthermore, silencing FOXK1 resulted in heightened radiosensitivity by impeding DNA damage repair, inducing G1 arrest, and promoting apoptosis. Subsequent studies demonstrated that FOXK1 directly bound to the promoter regions of CDC25A and CDK4, thereby activating their transcription in ESCC cells. Moreover, the biological effects mediated by FOXK1 overexpression could be reversed by knockdown of either CDC25A or CDK4. Collectively, FOXK1, along with its downstream target genes CDC25A and CDK4, may serve as a promising set of therapeutic and radiosensitizing targets for ESCC.

## Introduction

Esophageal cancer is the seventh most common cancer worldwide and the sixth leading cause of cancer-related mortality^1^. There are two main histological subtypes: esophageal squamous cell carcinoma (ESCC) and esophageal adenocarcinoma, with ESCC being the predominant type and having high morbidity and familial clustering in northern China^2,3^. The aggressive nature of ESCC with early loco-regional spread often leads to an advanced stage and poor prognosis. The standard therapy for patients with locally advanced disease is to receive definitive chemoradiation therapy (CRT) or neoadjuvant CRT followed by surgery. Although aggressive multimodal therapy has improved long-term survival in patients with locally advanced ESCC, the rate of local recurrence after CRT remains dismal. This may be attributed, in part, to the presence of intrinsic or radiation-induced resistant tumor cells, and the harmful effects of radiation on surrounding tissues^4–7^. However, efforts to enhance the prognosis by increasing the total dose of radiation or chemotherapy have shown limited success, since they may cause more treatment-related adverse effects^8,9^. Novel treatment modalities in conjunction with CRT are required to achieve superior local control rates, such as identification of ESCC-specific targeted agents, enhancement of radiosensitivity in ESCC, or interference with resistance mechanisms. Therefore, understanding the molecular drivers of tumorigenesis and radioresistance, and thus developing promising diagnostic and therapeutic strategies in ESCC remain a critical unmet clinical need.

FOX proteins are a superfamily of evolutionarily conserved transcriptional regulators that control a wide spectrum of biological processes^10^. As an important member of the FOX family, FOXK1 is also involved in the regulation of a variety of biological processes, including cell proliferation, differentiation, cell cycle progression, autophagy, glycolysis, and DNA damage response^11–16^. In addition, FOXK1 has been found to be upregulated in many malignant tissues and induces tumor proliferation and metastasis, and the oncogenic roles may be partly due to its involvement in the positive regulation of the cell cycle as well as its induction of EMT^17–20^. In particular, FOXK1 has been shown to promote proliferation and invasion of ESCC cells^21^. Nevertheless, the downstream target genes of FOXK1 and the mechanisms behind its oncogenic effects remain largely unknown. Furthermore, given that radiosensitivity is closely related to cell cycle position and cell cycle progression along with efficiency of DNA repair mechanisms, the role of FOXK1 as a master regulator of tumor cell cycle and DNA damage repair intrigues us to validate it as a potential target for enhancing radiosensitivity^22,23^.

In order to verify the biological effects and mechanism of FOXK1 in ESCC, in the present study, we assessed the expression of FOXK1 in ESCC cell lines and tissues, examined its function in regulating oncogenesis and radiosensitivity in ESCC cells, and analyzed the target genes of FOXK1 and their roles in mediating ESCC progression and response to radiation.

## Results

### Increased expression of FOXK1 in ESCC cell lines and tissues

**Figure 1:**
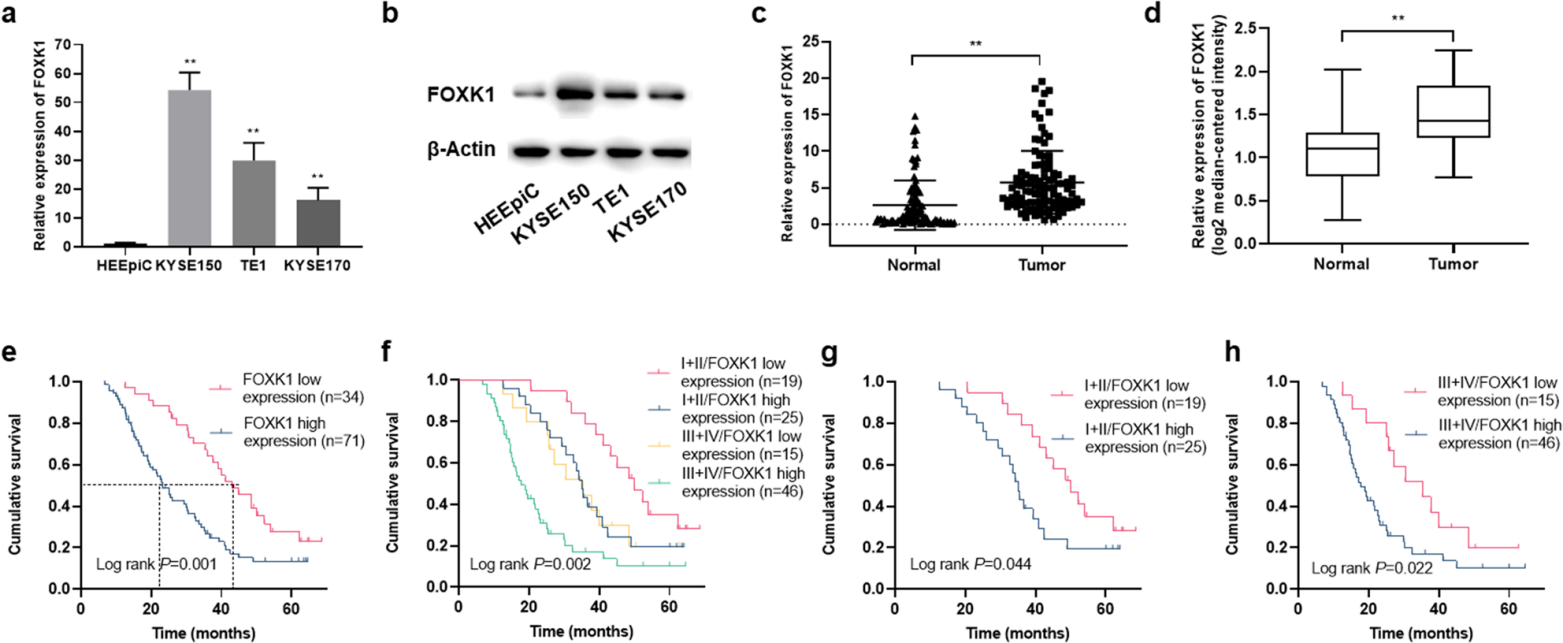
Expression status and survival analysis of FOXK1 in ESCC. (**a**, **b**) Expression of FOXK1 in HEEpiC cells and ESCC cells (KYSE150, TE1, and KYSE170) detected by qRT-PCR (**a**) and Western blot assays (**b**). Original western blots were presented in Supplementary Fig. 6a, with blots cut prior to hybridization with antibodies. (**c**) Expression of FOXK1 in 105 pairs of ESCC tissues and adjacent normal tissues detected by qRT-PCR method. (**d**) Relative expression levels of FOXK1 in ESCC tissues and corresponding normal esophageal tissues in the Oncomine database (n = 51) (Su Esophagus 2 Statistics). (**e**) The effect of expression levels of FOXK1 on survival of ESCC patients. (**f**) The effect of FOXK1 expression on survival rates of ESCC patients at different TNM stages. (**g**, **h**) The impact of FOXK1 expression on survival of stage I\II (**g**) and stage III/IV (**h**) ESCC patients. Log‐rank *P* < 0.05. The columns show the means of three independent experiments performed in triplicate, and the error bars indicate the standard deviation. Statistical significance was determined as *P < 0.05 and **P < 0.01.

The mRNA and protein expression levels of FOXK1 were significantly upregulated in three ESCC cells compared to normal esophageal epithelial cells, as detected by qRT-PCR and western blot assays (Fig. 1a,b). Upregulation of FOXK1 was also observed in ESCC tissues compared to corresponding adjacent normal tissues (*P* < 0.01) (Fig. 1c), and the same expression trend could be observed in the Oncomine database (Fig. 1d). According to the clinicopathological features, high expression levels of FOXK1 (the expression level of FOXK1 in tumor tissues was higher than that in corresponding normal tissues by 200%) were associated with TNM stage, invasion depth, and lymph node metastasis (Supplementary Table 1).

### Association between expression of FOXK1 and survival of ESCC patients

The impact of FOXK1 on the prognosis of ESCC patients was further evaluated. ESCC patients with low expression levels of FOXK1 (34/105, 32.4%) had better survival rates than those with high expression levels of FOXK1 (71/105, 67.6%) (*P* = 0.001) (Fig. 1e). The 5-year survival rate for patients with high expression levels of FOXK1 was 13.3% (median survival time, 23.2 months), while those with low expression levels of FOXK1 was 27.6% (median survival time, 43.2 months) (*P* = 0.001). A comprehensive analysis of the effects of clinical stage and FOXK1 expression on patients' prognosis revealed that stage III and IV patients with high expression levels of FOXK1 had poorer survival rates (Fig. 1f). Patients with stage I + II and stage III + IV were analyzed separately, and the patients with high expression levels of FOXK1 had significantly lower survival times than those with low expression levels of FOXK1 (*P* < 0.05), no matter in stage I + II or stage III + IV (Fig. 1g,h). Based on the results of univariate analysis, the significant prognostic factors for the survival of ESCC patients were TNM stage (*P* < 0.001), depth of invasion (*P* < 0.001), lymph node metastasis (*P* = 0.005), distant metastasis (*P* < 0.001), and FOXK1 expression (*P* = 0.001). The results of multivariate analysis showed that depth of invasion (*P* < 0.001), lymph node metastasis (*P* = 0.045), distant metastasis (*P* < 0.001), and FOXK1 expression (*P* = 0.003) were independently associated with the survival of ESCC patients (Supplementary Table 2).

### FOXK1 promotes ESCC cells proliferation, migration, and invasion in vitro

**Figure 2:**
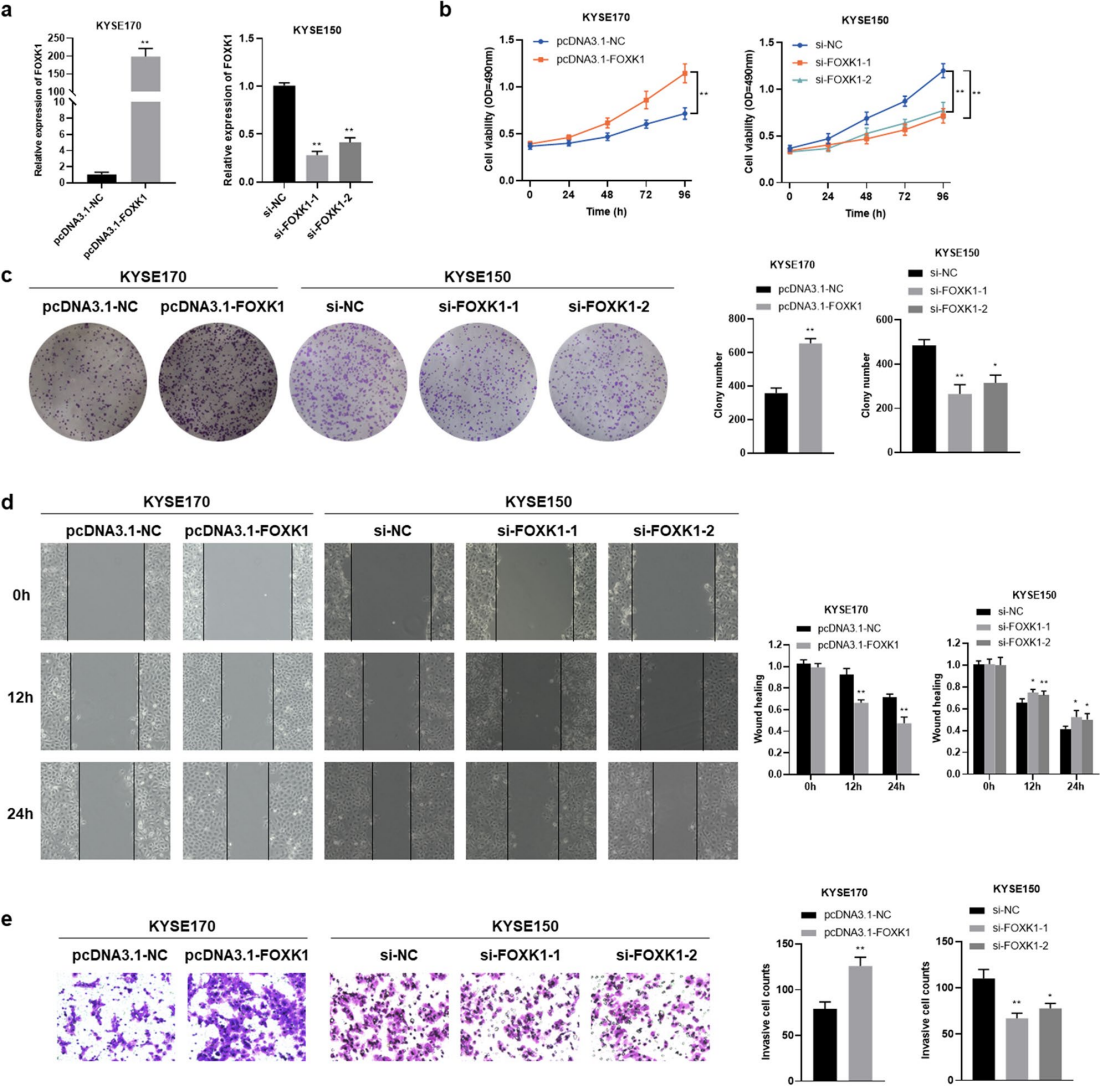
FOXK1 promotes proliferation, migration, and invasion of ESCC cells. (**a**) Transfection efficiency of FOXK1 overexpression in KYSE170 cells and knockdown in KYSE150 cells, examined by qRT-PCR assays. (**b**, **c**) MTS (**b**) and Colony formation (**c**) assays were used to detect cell proliferation of FOXK1‐overexpressing KYSE170 cells and FOXK1-knockdown KYSE150 cells, respectively. (**d**, **e**) Wound healing (**d**) and transwell (**e**) assays were performed to detect cell migration and invasion ability in FOXK1-overexpressing KYSE170 cells and FOXK1-knockdown KYSE150 cells. Values represent the Mean ± SD for three independent experiments. *P < 0.05, **P < 0.01.

We investigated the effect of FOXK1 on ESCC cell proliferation, migration, and invasion. Considering the expression level of FOXK1 in ESCC cell lines, the pcDNA3.1-FOXK1 plasmid was used to overexpress FOXK1 in KYSE170 and TE1 cells, and siRNAs (si-FOXK1-1 and si-FOXK1-2) were used to knockdown FOXK1 in KYSE150 and TE1 cells. The transfection efficiency was verified by qRT-PCR assays (Fig. 2a, Supplementary Fig. 1a). Overexpression of FOXK1 enhanced the proliferation, migration, and invasion ability of KYSE170 and TE1 cells (Fig. 2b–e, Supplementary Fig. 1b–e). In contrast, knockdown of FOXK1 significantly inhibited proliferation, migration, and invasion ability of KYSE150 and TE1 cells (Fig. 2b–e, Supplementary Fig. 1b–e).

### FOXK1 confers radioresistance and regulates the phosphorylation of H2AX after irradiation

**Figure 3:**
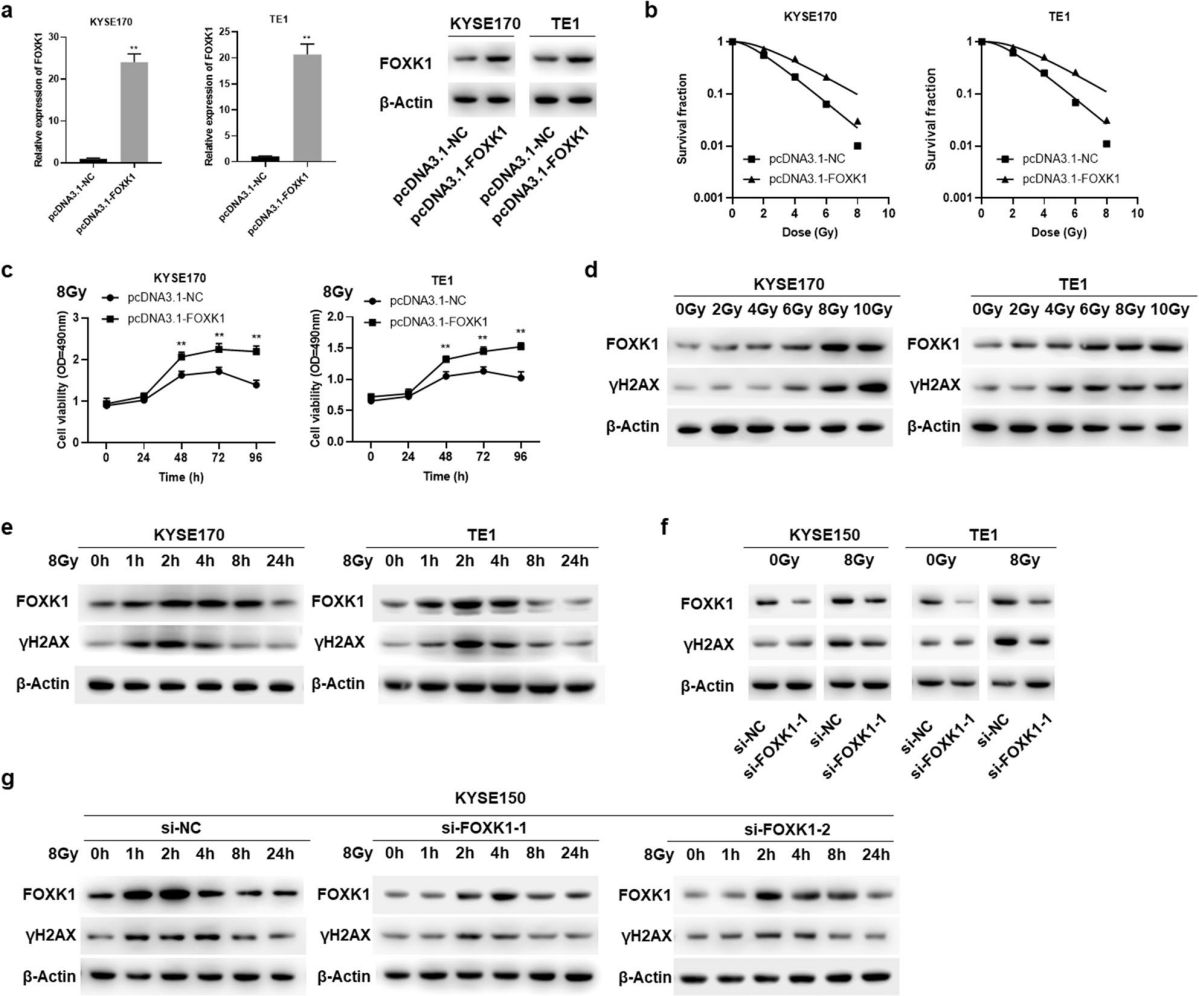
Radiosensitivity analysis of FOXK1 in ESCC cells. (**a**) Stable transfection efficiency of FOXK1 overexpression in KYSE170 and TE1 cells, detected by qRT-PCR and Western blot assays, respectively. Original western blots were presented in Supplementary Fig. 6b, with blots cut prior to hybridization with antibodies. (**b**) Colony formation assays were used to explore the influence of overexpression of FOXK1 on clonal survival of KYSE170 and TE1 cells following graded doses of irradiation. (**c**) The effect of upregulation of FOXK1 on the viability of KYSE170 and TE1 cells at different times after radiation exposure was examined by MTS assays. (**d**) The protein expression levels of FOXK1 and γH2AX in KYSE170 and TE1 cells after graded doses of irradiation. Original western blots were shown in Supplementary Fig. 6c, with blots cut prior to hybridization with antibodies. (**e**) The protein expression levels of FOXK1 and γH2AX in KYSE170 and TE1 cells at different time points after irradiation. Original western blots were presented in Supplementary Fig. 6d. (**f**) The effect of silencing FOXK1 on the protein expression of γH2AX in irradiated and non-irradiated KYSE150 and TE1 cells. Original western blots were displayed in Supplementary Fig. 6e. (**g**) Protein expression levels of FOXK1 and γH2AX in FOXK1 knockdown KYSE150 cells at different time points after irradiation. Original western blots were shown in Supplementary Fig. 6f, with blots cut prior to hybridization with antibodies. Data presented are the mean ± SD, from three independent experiments. A representative data from three independent experiments is shown. *P < 0.05, **P < 0.01.

We analyzed whether FOXK1 affected the radiosensitivity of ESCC cells. The expression level of FOXK1 was significantly upregulated in pcDNA3.1-FOXK1 stably transfected KYSE170 and TE1 cells (Fig. 3a). According to the results of colony formation assays, cells with upregulated FOXK1 showed increased clonal survival after radiation exposure, and their survival curves showed higher D0, N, and Dq values (*P* < 0.05), indicating that overexpression of FOXK1 conferred radioresistance (Fig. 3b, Supplementary Table 3). The cell viability of FOXK1 overexpressing ESCC cells after irradiation was also significantly higher than that of the corresponding control cells (Fig. 3c). We next assessed whether FOXK1 was involved in radiation-induced DNA damage repair. ESCC cells were treated with graded doses of radiation (0–10 Gy) and incubated for 3 h before harvesting. As shown in Fig. 3d, ionizing radiation (IR) activated expression of FOXK1 and γH2AX in a dose-dependent manner in KYSE170 and TE1 cells. To detect the time course of induction of FOXK1 and γH2AX after irradiation, the cells were exposed to 8 Gy radiation and harvested after different time intervals. Induction of FOXK1 and γH2AX was significantly increased in cells harvested after irradiation within 1 h, and their protein levels reached a maximum at 2 h, and then started declining gradually after 4 h (Fig. 3e). Subsequently, we exposed FOXK1 knockdown KYSE150 and TE1 cells to radiation to test the expression of γH2AX. As displayed in Fig. 3f, the expression of γH2AX remained almost unchanged in FOXK1 knockdown ESCC cells when cells were not exposed to irradiation. However, downregulation of FOXK1 significantly inhibited the activation of γH2AX when the cells were irradiated. We further exposed FOXK1 knockdown KYSE150 cells to 8 Gy radiation and extracted the proteins of FOXK1 and γH2AX at different time points after irradiation. Compared to the si-NC group, down-regulation of FOXK1 groups showed decreased expression levels of FOXK1 and γH2AX at all time points after irradiation. Additionally, peak expression levels of FOXK1 and γH2AX in the si-FOXK1-1 and si-FOXK1-2 groups occurred at 2–4 h after irradiation, which was later than that in the si-NC group which was at 2 h (Fig. 3g). These results suggested that knockdown of FOXK1 resulted in reduced and delayed expression of γH2AX in irradiated ESCC cells, thereby inhibiting DNA damage repair and suppressing radiosensitivity.

### Effect of knockdown of FOXK1 on cell cycle and apoptosis after irradiation of esophageal cancer cells

**Figure 4:**
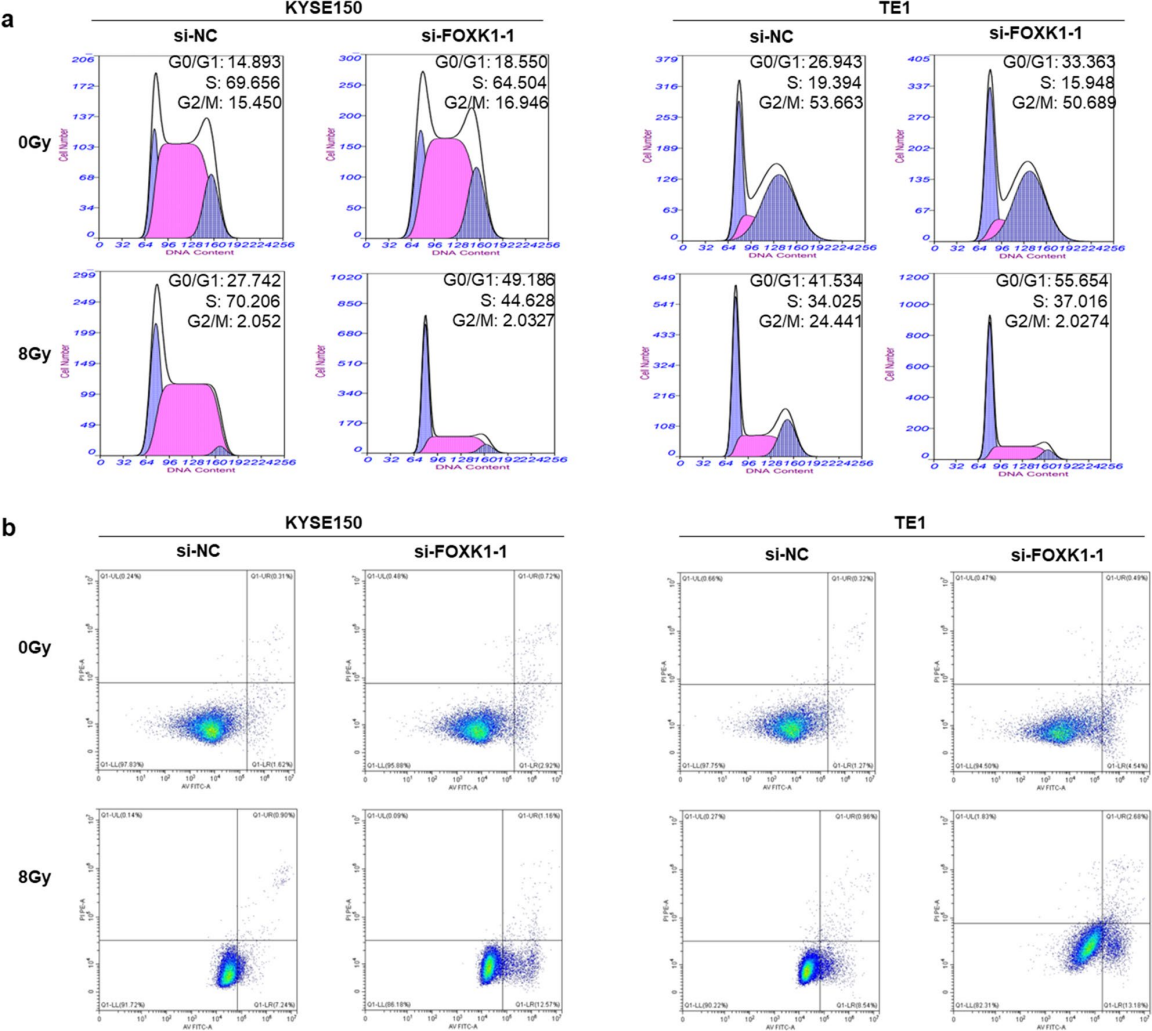
Effect of FOXK1 on cell cycle and apoptosis of ESCC cells after irradiation. (**a**) The flow cytometry was used to measure the cell cycle of FOXK1 knockdown KYSE150 and TE1 cells after irradiation or non-irradiation. (**b**) The effect of downregulating FOXK1 on the apoptosis rate of irradiated and non-irradiated KYSE150 and TE1 cells was detected by flow cytometry. A representative data from three independent experiments is shown.

Further investigations were conducted to determine if FOXK1 inhibited radiosensitivity through cell cycle redistribution and apoptosis. Knockdown of FOXK1 resulted in G1 arrest in KYSE150 and TE1 cells, whereas cells with knockdown of FOXK1 exhibited more pronounced G1 arrest after irradiation (Fig. 4a). Silencing FOXK1 also induced apoptosis and this effect was augmented by radiation exposure (Fig. 4b). Western blot analysis of cell cycle and apoptosis related proteins showed that cyclin D1, CDK4, and Bcl-2 were downregulated in FOXK1 knockdown cells, while p21 and Bax were upregulated, and these trends were more drastic in irradiated FOXK1 knockdown cells (Supplementary Fig. 2a,b). These findings demonstrated that the radiosensitizing effect of FOXK1 knockdown was contributed to G1 arrest and apoptosis.

### FOXK1 increases the transcription of CDC25A and CDK4 by directly binding to their promoters

**Figure 5:**
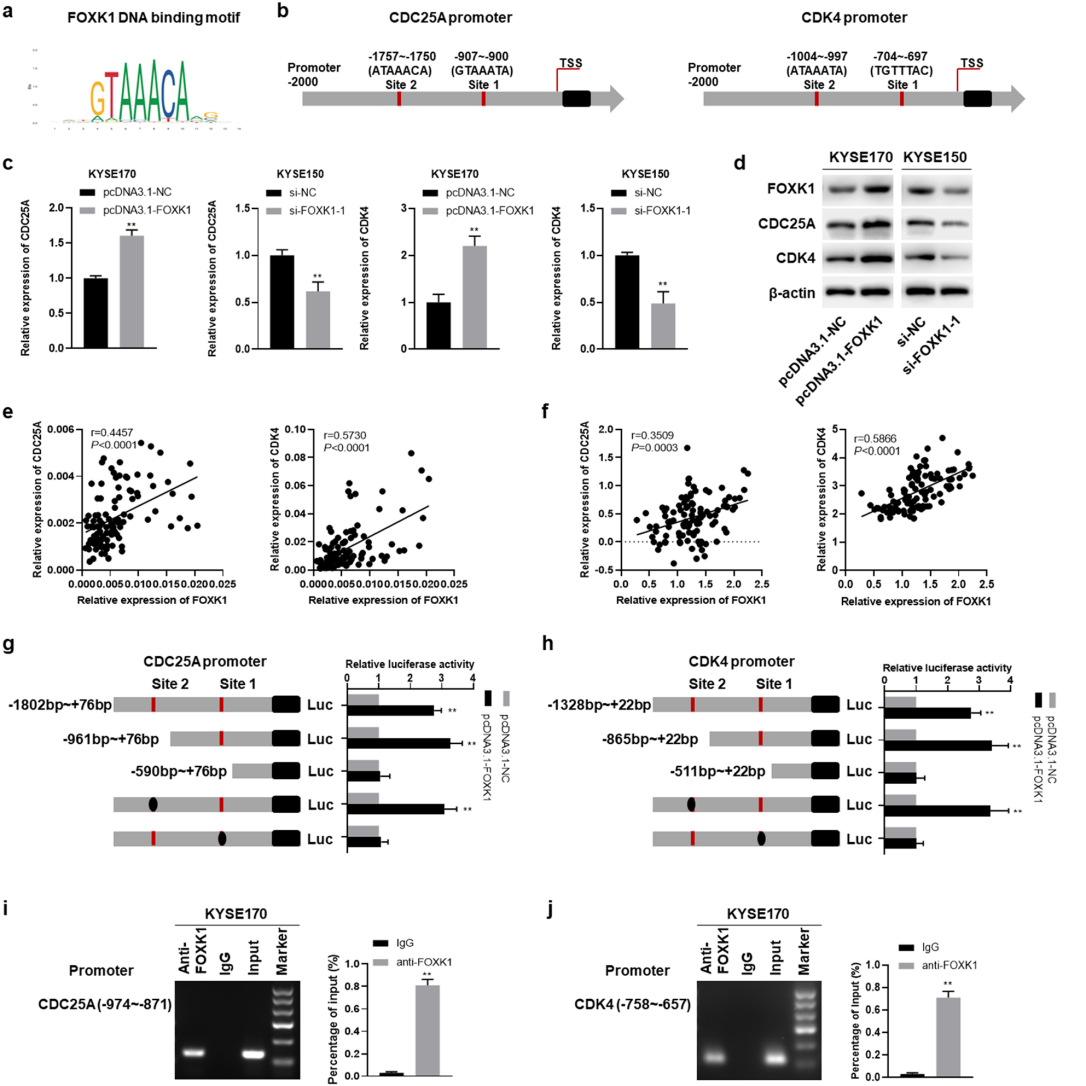
FOXK1 increases the transcription of CDC25A and CDK4 through direct binding to their promoter. (**a**) The motif of FOXK1 was analyzed by JASPAR. (**b**) The putative FOXK1 binding sites in the proximal promoter regions of CDC25A and CDK4. (**c**, **d**) The relative mRNA (**c**) and protein (**d**) expression of CDC25A and CDK4 in FOXK1-upregulated KYSE170 cells and FOXK1-downregulated KYSE150 cells, examined by qRT-PCR and Western blot assays, respectively. Original western blots were presented in Supplementary Fig. 6g, with blots cut prior to hybridization with antibodies. (**e**) The correlation between relative mRNA expression of FOXK1 and its target genes CDC25A and CDK4 in ESCC tissues (n = 105). (**f**) Relative expression of FOXK1 in relation to CDC25A and CDK4, retrieved from the Oncomine database. (**g**, **h**) The binding sites of FOXK1 to the CDC25A (**g**) and CDK4 (**h**) promoters were identified by dual luciferase reporter assays. (**i**, **j**) ChIP experiments confirmed the enrichment of FOXK1 on the promoters of CDC25A (**i**) and CDK4 (**j**). Data presented are the mean ± SD from at least three independent experiments. *P < 0.05, **P < 0.01.

The FOXK1 binding motif was retrieved from JASPAR^24^ (Fig. 5a). A set of proliferation-related genes containing this motif in their promoter regions were screened, including CCND3, CCNE2, CDC7, CDC20, CDC23, CDC25A, CDC25C, CDK1, CDK4, FGF12, FGFR2, FGFR4, NOTCH1, AKT2, BMP6, BMP7, ROCK1, TGFB1, TGFBR2, VEGFA, PDGFRA, DVL2, DVL3, FZD1, FZD3, and FZD6. Among these genes, CCNE2, CDC25A, CDK4, AKT2, and FZD3 demonstrated an increase trend in FOXK1 overexpressed KYSE170 and TE1 cells, and a decrease trend in FOXK1 knockdown KYSE150 and TE1 cells (Supplementary Fig. 3a,b). Among them, we focused on CDC25A and CDK4, which were involved in tumor growth and radiosensitivity^25–28^. As shown in Fig. 5b, two putative FOXK1 binding sites were respectively identified around the TSS regions of CDC25A and CDK4. Overexpression of FOXK1 increased the mRNA and protein expression levels of CDC25A and CDK4, whereas silencing FOXK1 suppressed their expression (Fig. 5c,d). The expression levels of CDC25A and CDK4 in ESCC tissues were also positively correlated with FOXK1 (Fig. 5e), and similar trends could be observed in the Oncomine database (Fig. 5f).

Next, a series of deletion constructs of the CDC25A and CDK4 promoters were subcloned into the pGL3-Basic vector. As shown in Fig. 5g, the plasmids pCDC25A-1802/+ 76 and pCDC25A-961/+ 76 exhibited higher luciferase activity in FOXK1 overexpressing KYSE170 cells, whereas the pCDC25A-590/+ 76 showed almost no luciferase activity. To identify the specific binding site, we mutated site 1 (− 907 bp to − 900 bp) and site 2 (− 1757 bp to − 1750 bp) separately. The luciferase activity showed a significant decrease when site 1 was mutated, while mutation at site 2 showed no marked change, indicating the critical role of site 1. For the CDK4 promoter, the deletion constructs pCDK4-1328/+ 22 and pCDK4-865/+ 22 displayed substantially increased luciferase activity in FOXK1 overexpressing KYSE170 cells, however, the FOXK1-dependent luciferase activity failed to elevate in pCDK4-511/+ 22. Moreover, only mutation in site 1 (− 704 bp to − 697 bp) decreased the luciferase activity, indicating that this may be the binding site for FOXK1 to CDK4 promoter (Fig. 5h). Further ChIP assays confirmed the enrichment of FOXK1 at site 1 of the CDC25A promoter and also at site 1 of the CDK4 promoter (Fig. 5i,j). These results collectively suggested that the elevated expression of CDC25A and CDK4 in ESCC might be partly due to the transcriptional regulation of FOXK1.

### Overexpression of FOXK1 enhances ESCC cells growth, migration, and invasion through regulation of CDC25A and CDK4

**Figure 6:**
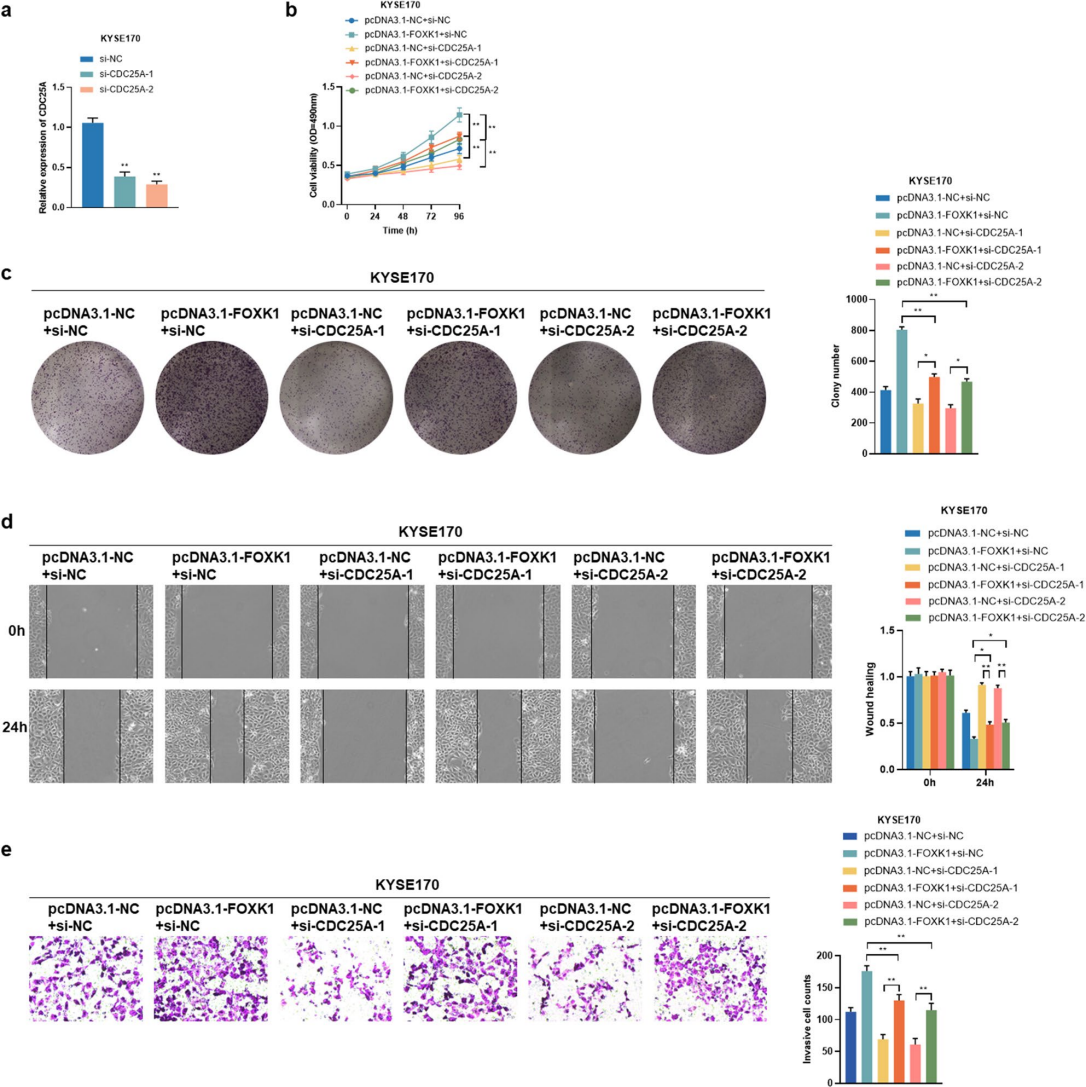
FOXK1 promotes proliferation, migration and invasion of ESCC cells through upregulation of CDC25A. (**a**) Transfection efficiency of CDC25A knockdown in KYSE170 cells, examined by qRT-PCR assays. (**b**, **c**) The effect of knockdown of CDC25A on the proliferative capacity of FOXK1 overexpressing KYSE170 cells was examined using MTS (**b**) and colony formation (**c**) assays. (**d**, **e**) The migration and invasion were confirmed by wound healing (**d**) and transwell (**e**) assays after silencing CDC25A in FOXK1 overexpressing KYSE170 cells. Data presented are the mean ± SD from at least three independent experiments. *P < 0.05, **P < 0.01.

**Figure 7:**
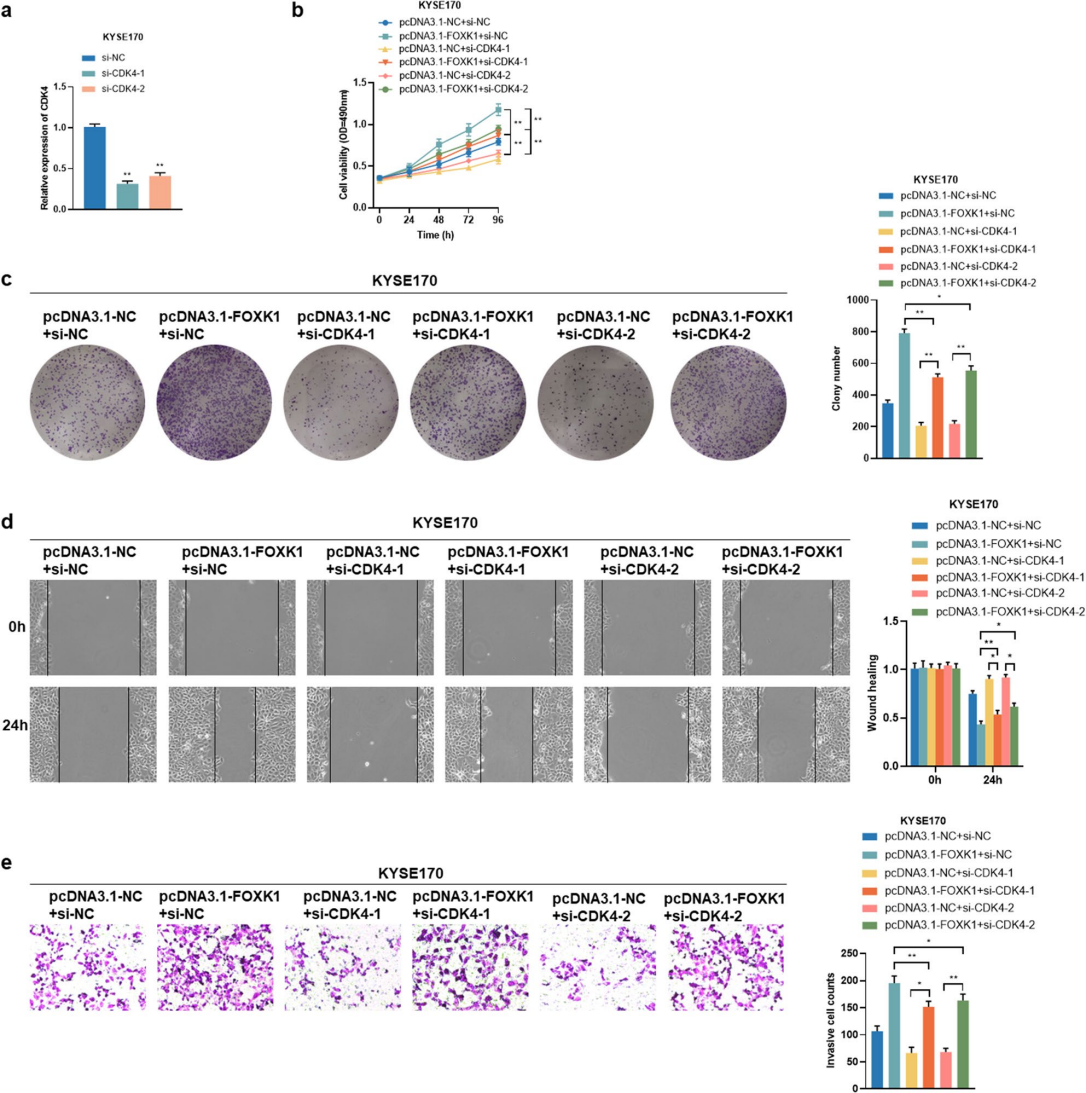
FOXK1 stimulates proliferation, migration and invasion of ESCC cells through upregulation of CDK4. (**a**) Transfection efficiency of CDK4 knockdown in KYSE170 cells, examined by qRT-PCR assays. (**b**, **c**) The impacts of knockdown of CDK4 on the proliferation capacity of KYSE170 cells overexpressing FOXK1 were verified by MTS (**b**) and colony formation (**c**) assays. (**d**, **e**) The effects of CDK4 deficiency on migration and invasion of FOXK1 overexpressing KYSE170 cells were examined using wound healing (**d**) and transwell (**e**) assays. Data presented are the mean ± SD from at least three independent experiments. *P < 0.05, **P < 0.01.

To verify whether FOXK1 exerted oncogenic effects in ESCC cells by controlling the transcription of CDC25A and CDK4, we further performed rescue experiments by knocking down CDC25A and CDK4, respectively, in FOXK1 overexpressing KYSE170 and TE1 cells. The transfection efficiency of knocking down CDC25A with si-CDC25A-1 and si-CDC25A-2 in KYSE170 and TE1 cells was verified by qRT-PCR assays (Fig. 6a, Supplementary Fig. 4a). As shown in Fig. 6b–e and Supplementary Fig. 4b–e, the reduction of CDC25A partially reversed the enhanced proliferation, migration, and invasion ability induced by overexpression of FOXK1. Transfection with both si-CDK4-1 and si-CDK4-2 could significantly knock down CDK4 in KYSE170 and TE1 cells (Fig. 7a, Supplementary Fig. 5a). As illustrated in Fig. 7b–e and Supplementary Fig. 5b–e, silencing CDK4 also attenuated the role of FOXK1 in promoting ESCC cells proliferation, migration, and invasion. Together, it can be concluded that FOXK1 played an oncogenic role in ESCC by transcriptional activation of CDC25A and CDK4.

### Overexpression of FOXK1 confers radioresistance by activating CDC25A and CDK4 in ESCC cells

**Figure 8:**
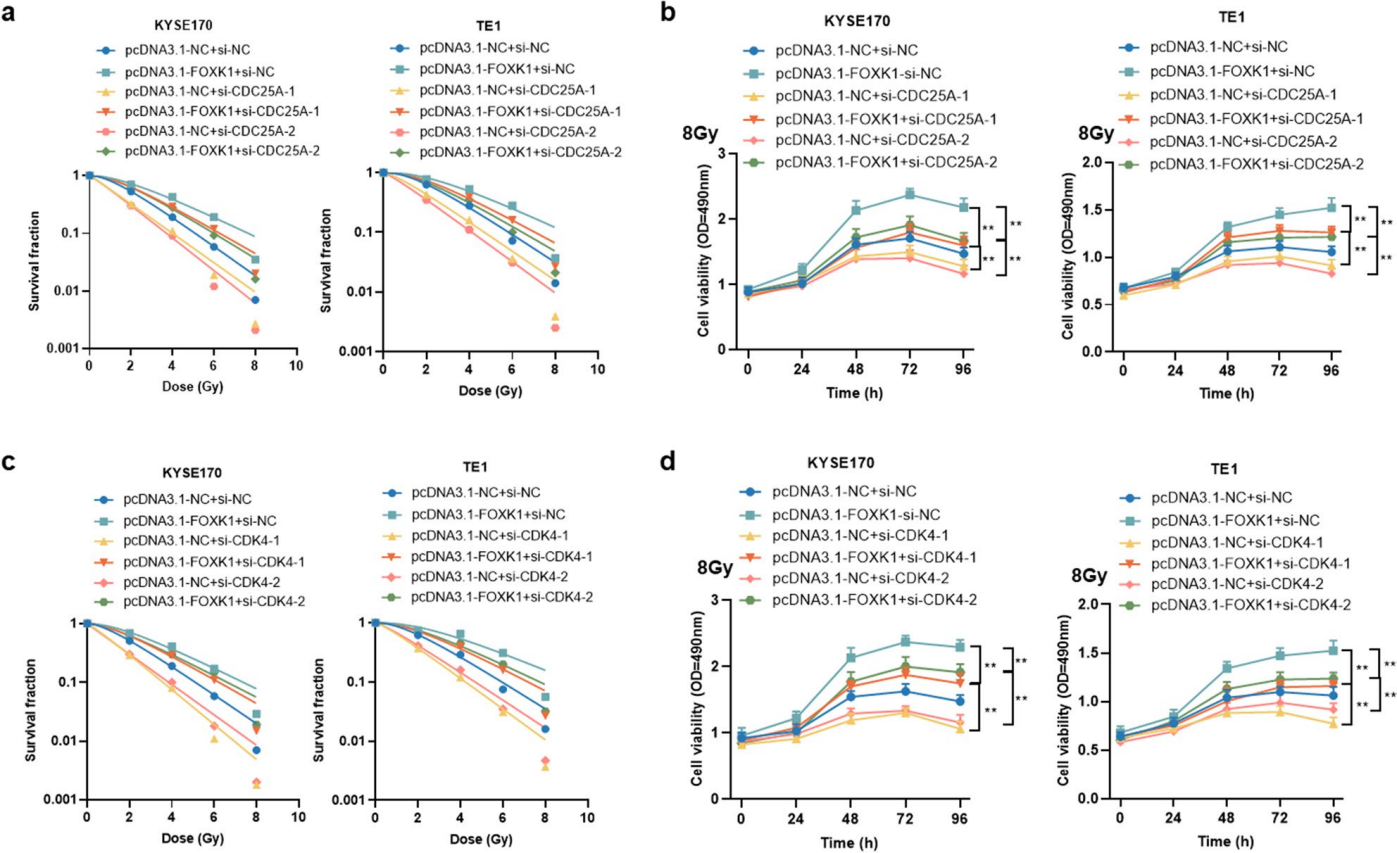
FOXK1 attenuates radiosensitivity of ESCC cells through upregulation of CDC25A and CDK4. (**a**) The radiosensitivity was tested by colony formation assays after downregulation of CDC25A in FOXK1 overexpressing KYSE170 and TE1 cells. (**b**) The influence of irradiation on cell viability was determined by MTS assays following knockdown of CDC25A in FOXK1 overexpressing KYSE170 and TE1 cells. (**c**) Cellular radiosensitivity was examined by colony formation assays after knockdown of CDK4 in FOXK1 overexpressing KYSE170 and TE1 cells. (**d**) The effect of irradiation on cell viability was ascertained using MTS assays after silencing CDC25A in FOXK1 overexpressing KYSE170 and TE1 cells. Data presented are the mean ± SD from at least three independent experiments. *P < 0.05, **P < 0.01.

To further validate the involvement of CDC25A and CDK4 in FOXK1-regulated radiosensitivity of ESCC cells, rescue experiments were performed. The survival fraction of KYSE170 and TE1 cells gradually decreased with increasing radiation dose, while FOXK1 overexpressing cells exhibited relatively higher clonal survival. However, knockdown of CDC25A inhibited this proliferative capacity of FOXK1 (Fig. 8a). By assessing cell viability at different time points after irradiation, the cell viability of FOXK1 overexpressing cells was significantly increased compared to the corresponding control cells; while downregulation of CDC25A suppressed the increase in viability induced by FOXK1 (Fig. 8b). Similarly, CDK4 deficiency attenuated the clonal proliferation capacity of FOXK1 overexpressing cells after graded doses of radiation (Fig. 8c). Silencing CDK4 also made FOXK1 overexpressing ESCC cells less capable of proliferation at different time points after irradiation (Fig. 8d). These results supported that FOXK1-mediated radioresistance of ESCC cells might depend on its transcriptional regulation of CDC25A and CDK4.

## Discussion

The FOX proteins play pleiotropic roles in biological processes, and their dysfunction is associated with tumorigenesis and cancer progression^10,29^. FOXK1, an important member of the FOX family, is located at chromosomal position 7p22. FOXK1 has been proved to play an oncogenic role in a variety of cancers; however, its role and molecular mechanisms in the progression and radiosensitivity of esophageal cancer are not well clarified. In the present study, upregulation of FOXK1 was detected in ESCC cells and tissues, which was associated with tumor stage and poor prognosis. In addition, FOXK1 enhanced proliferation, migration, invasion, and radiation resistance of ESCC cells through transcriptional upregulation of CDC25A and CDK4.

Upregulation of FOXK1 has been detected in several types of human cancers including gastric, gallbladder, and colorectal cancers, and high expression of FOXK1 may lead to poor prognosis in different types of cancers^18,20,30^. Previous study has shown elevated expression of FOXK1 in ESCC tissues, which affects the survival of ESCC patients^21^. Our study and Chen’s study^21^ also demonstrated the promoting effects of FOXK1 on ESCC cell proliferation, migration, and invasion, which were consistent with the studies in hepatocellular carcinoma, gastric cancer, colorectal cancer, and gallbladder cancer^18–20,31^. These results suggest that FOXK1 may play an oncogenic role in multiple malignancies, including ESCC.

Studies have shown that FOXK1 has a role in regulating DNA damage repair^16^, which inspired us to investigate its effect on radiosensitivity. Our results suggested that FOXK1 conferred radioresistance to ESCC cells. IR is known to induce cell death mainly by causing DNA double-strand breaks (DSBs). The early cellular response to DSBs is C-terminal phosphorylation of H2AX (termed γH2AX), and subsequently γH2AX serves as a platform to recruit other DNA repair proteins to the site of DSBs^32,33^. Therefore, γH2AX is widely used as a marker for detecting radiation-induced DSBs repair^34,35^. In the present study, FOXK1 promoted the activation of γH2AX after irradiation, suggesting that FOXK1 may confer radioresistance by inducing DNA damage repair. In addition, IR exposure causes cells to arrest in G1 and G2 phases, and cells possessing G1 arrest are more sensitive to IR^36,37^. Consistent with these reports, our observation of FOXK1 deficiency promoting G1 arrest in irradiated cells suggests its role in radiosensitivity. Targeting FOXK1 may be a useful adjunct to radiation therapy in the treatment of ESCC.

To further elucidate the molecular mechanism of FOXK1, we identified two novel FOXK1 target genes in ESSC: CDC25A and CDK4. CDC25A is a member of the CDC25 family of phosphatases. It mainly activates CDK2 and thereby induces progression in the cell cycle from G1 to S phase^38^. The upregulation of CDC25A in a variety of tumors and its oncogenic role have been widely reported^39,40^. CDC25A has also been shown to modulate radiosensitivity in esophageal, cervical, and lung cancers^26,41,42^. Another FOXK1 target gene, CDK4, is a catalytic subunit of the protein kinase complex that plays a critical role in the G1-S checkpoint^43^. CDK4 has functions in promoting proliferation, migration, invasion, and angiogenesis in various cancers^27,44^. Moreover, CDK4/6 inhibitors have had great success in treating certain cancer types, such as breast and lung cancer, and are in clinical trials for many other tumors^45–48^. As silencing CDK4 resulted in the blockade of cell cycle progression, enhancement of apoptosis, and suppression of DNA damage repair, CDK4 has recently been proved to be a novel target for radiosensitization^49–51^. Particularly, several preclinical studies have validated the radiosensitization effects of CDK4/6 inhibitors in vitro and in vivo^52,53^. Our study is consistent with these findings and suggests a critical role for both CDC25A and CDK4 in oncogenicity and radioresistance of ESCC cells, which is transcriptionally controlled by FOXK1.

In conclusion, our study revealed the upregulation of FOXK1 in ESCC and its adverse effect on prognosis. FOXK1 exerts oncogenic and radioresistant role through transcriptional activation of CDC25A and CDK4. FOXK1 may act as a promising biomarker and a novel therapeutic target for ESCC.

## Materials and methods

### Patients and tissue specimens

A total of 105 cases of ESCC tissues and paired adjacent non-tumor tissues were obtained from patients admitted to the Fourth Hospital of Hebei Medical University between 2012 and 2016 with a confirmed diagnosis of ESCC who received surgical resection as their first treatment. Each surgically excised specimen was placed in liquid nitrogen immediately after acquisition and stored at − 80 °C for RNA extraction. Treatment records, clinical history, and follow-up data of patients were retrieved from the case management system in the medical record room. Grading and staging were performed according to the 8th edition AJCC/UICC staging of cancers of the esophagus and esophagogastric junction. A positive family history of upper gastrointestinal cancer (UGIC) was defined as a self-reported history of esophageal/cardia/gastric cancer in at least one of the first-degree relatives or two second-degree relatives. Written informed consent was obtained from all patients, and the study was approved by the Ethics Committee of the Fourth Hospital of Hebei Medical University.

### Cell culture and X‑ray irradiation

Human esophageal cancer cell lines KYSE150, TE1, KYSE170 and human normal esophageal epithelial cell line HEEpiC were purchased from China Center for Type Culture Collection (CCTCC, Wuhan, China). The ESCC cell lines were cultured in RPMI 1640 (Gibco, Invitrogen, Life Technologies, Germany) medium supplemented with 10% heat-inactivated fetal bovine serum (FBS) (Invitrogen, Carlsbad, CA, USA) at 37 °C with 5% CO_2_. The HEEpiC cells were cultured according to the manufacturer’s instructions.

Irradiation was performed at room temperature with a 6-MV Varian linear accelerator (Varian Medical Systems, Palo Alto, CA, USA) at a dose rate of 5 Gy/min and a gantry angle of 180 degrees. Cells were cultured in six-well plates or cell culture dishes with a 1 cm tissue compensator placed underneath, and the source-skin distance was set to 100 cm. The radiation dose was a single 8 Gy, except for the experiments with graded-dose exposure. After irradiation, cells were recovered in respective growth medium for the indicated time until harvesting.

### RNA isolation and quantitative real-time polymerase chain reaction (qRT-PCR) assay

Total RNA was isolated from tissue samples and cell lines using TRIzol reagent (Invitrogen, Carlsbad, CA, USA), and the RNA was reverse transcribed into cDNA with Transcriptor First Strand cDNA Synthesis Kit (Roche, Basel, Switzerland) according to the manufacturer’s instructions. The qRT-PCR was performed in triplicate with StepOnePlus Real-Time PCR System (Applied Biosystems) using GoTap® qPCRMaster Mix (Promega, Madison, WI, USA). The expression of genes was normalized to the expression of GAPDH. Data were analyzed and presented using the 2^−ΔΔCt^ method. All primers used for qRT-PCRs were listed in Supplementary Table 4.

### Cell transfection

FOXK1 siRNAs, CDK4 siRNAs, and CDC25A siRNAs were purchased from Ribbio (Guangzhou, China). The siRNA sequences were shown in Supplementary Table 5. For overexpression of FOXK1, CDK4, and CDC25A, the cDNAs encoding them were PCR-amplified and subcloned into pcDNA3.1 vector (Invitrogen, Carlsbad, CA, USA). For transient transfection, Lipofectamine 2000 reagent (Invitrogen, Carlsbad, CA, USA) was used according to the manufacturer’s instructions. The cells were collected 48 h after transfection with siRNAs or plasmids. The infection efficiency was validated using qRT-PCR or western blot assays. To generate FOXK1 stably expressing cells, the cells were isolated using 800 μg/mL of G418 after transient transfection.

### Western Blot assay

Total protein extraction was performed using RIPA lysis buffer containing PMSF (Solarbio, Beijing, China). Protein concentration was determined using the BCA Protein Assay (Multi Sciences, Hangzhou, China). Equal amounts of protein samples were electrophoresed on 10% sodium dodecyl sulfate–polyacrylamide electrophoresis gel and then transferred onto polyvinylidene fluoride membranes (Millipore, Billerica, MA, USA), followed by blocking with 5% skim milk for an hour. Afterwards, the membranes were probed with specific primary antibodies overnight at 4 °C and then with secondary antibody (KPL, Milford, MA, USA) for an hour at room temperature. The primary antibodies used were listed in Supplementary Table 6. Finally, the signals were visualized using an enhanced chemiluminescence reagent (Multi Sciences, Hangzhou, China).

### Cell viability assay

Cell viability was assessed using an MTS (3-(4,5-dimethylthiazol-2-yl)-5-(3-carboxymethoxyphenyl)‐2‐(4‐sulfophenyl)‐2H‐tetrazolium) assay. The CellTiter 96® Aqueous One Solution Cell Proliferation Assay kit (Promega, Madison, WI, USA) was used according to the manufacturer's instructions. The transfected cells were seeded at 1 × 10^3^ cells/well and cultured in 96‐well plates. MTS reagent (20 µL) was added to 100 µl of culture medium at different time points and incubated for 2 h. Then, the cell viability was calculated by detecting the absorbance of each well at 490 nm via a Spark® multimode microplate reader (Tecan).

For cell viability after irradiation, cells were plated in 96-well plates at a density of 5 × 10^3^ cells/mL and incubated for 24 h at 37 °C. The viability of cells irradiated at 8 Gy was determined by incubation with MTS reagent, as described above. All experiments were repeated at least three times.

### Colony formation assay

The 5 × 10^3^ transfected cells were inoculated in triplicate in 6‐well plates and cultured for approximately 7 days until colonies were visible. The colonies (containing 50 cells or more) were manually counted after fixation with 4% paraformaldehyde and staining with 0.1% crystal violet.

For the colony formation assay after irradiation, cells stably expressing FOXK1 and their respective control cells were plated at a low density onto 6-well plates (500 cells/well), followed by graded single doses (0–8 Gy). Two weeks later, the cells were fixed and stained with crystal violet (0.6%). Colonies of ≥ 50 cells were scored as survivors. The mean survival data for each individual cell line were fitted to the linear quadratic (LQ) model: SF = 1 − (1 − e^−D/D0^)^N^, where SF is the survival fraction, D is the irradiation dose, D0 is the mean lethal dose, and N is the extrapolation number.

### Wound-healing assay

Cell migration capacity was evaluated using wound healing assays. The transfected cells were seeded into 6-well plates and allowed to grow to confluence, after which they were starved with serum-free medium. A scratch wound was generated using a sterile 200 μL pipette tip, and floating cells were removed by washing with 1 × PBS. Migration was photographed at indicated time points after scratching using an inverted microscope, and the percentage of wound closure was calculated for three randomly chosen fields.

### Cell invasion assay

Cell invasion assays were performed in Matrigel-coated chambers (Corning Costar, Corning, NY, USA) with 8-μm pore membranes. The 1 × 10^5^ transfected cells suspended in 200 μL of serum-free RPMI 1640 medium were added to the upper chamber while the lower compartment of the chamber was filled with 600 μL of the medium containing 10% FBS. After 24 h of incubation at 37 °C, the non-migrating cells in the upper chamber were removed by a cotton swab and lower surface of the chamber was fixed with 4% paraformaldehyde and stained with crystal violet. Invading cells were scored by counting at least 5 fields per membrane under a light microscope.

### Flow cytometry analysis of cell cycle and apoptosis

FITC Annexin V and propidium iodide (PI) (BD bioscience, CA, USA) double staining was used to detect cell apoptosis, while PI (Multi Science, Hangzhou, China) mono staining was applied to monitor cell cycle. After harvesting the transfected cells with or without 8 Gy single-dose of irradiation, the cell concentration was adjusted to 1 × 10^6^ cells per mL and then washed twice with PBS. Thereafter, apoptosis was detected by adding 5 μL of FITC Annexin V and 5 μL of PI to 100 μL of cell solution for staining in the dark at room temperature for 15 min. To detect cell cycle, the cells were stained with 500 μL of PI (1 μg/mL) for 30 min at room temperature in the dark. The flow cytometer was used to detect cell apoptosis and cell cycle.

### Dual-luciferase reporter assays

The CDC25A promoter construct was obtained from PCR amplified fragment from genomic DNA, and was cloned into the pGL3-Basic vector. KYSE170 cells overexpressing FOXK1, KYSE150 cells with knockdown of FOXK1, and their respective control transfectants were plated at a density of 1 × 10^5^ to 5 × 10^5^ cells/mL into 24-well plates and transfected with 0.2 μg pGL3-CDC25A promoter luciferase plasmid and 0.02 μg of the SV40 Renilla luciferase plasmid. After 48 h, firefly and Renilla luciferase activities were assayed sequentially in each well using the Dual-Luciferase Reporter Assay System (Promega, Madison, WI, USA). Transcriptional activity was calculated as the ratio of firefly luciferase activity (reporter) to Renilla luciferase activity (control). All luciferase assays were carried out in triplicate. The CDK4 promoter construct was generated in a similar manner, and dual-luciferase reporter assays were conducted as described above. The primers for a series of CDC25A and CDK4 promoter fragments were listed in Supplementary Table 7.

### Chromatin immunoprecipitation (ChIP) assay

ChIP assays were performed using EZ-Magna ChIP A/G (17-10086, Upstate, Millipore, MA, USA) kit to study the enrichment of FOXK1 in CDK4 and CDC25A promoter regions according to the manufacturer’s instructions. Antibody against FOXK1 (ab85999; Abcam) was used for immunoprecipitation. Quantitative analysis of ChIP-derived DNA was performed by real-time qPCR analysis (primers in Supplementary Table 7).

### Statistical analysis

Statistical analyses were conducted with GraphPad Prism 8.0 and SPSS 25.0. Student’s t-test or Pearson's χ^2^ test was used to determine statistical significance. Error bars show mean ± standard deviation (SD) of independent experiments. All tests were performed two-tailed and *P* values < 0.05 were considered statistically significant. Kaplan–Meier analyses were used for overall survival curves and *P* values were calculated with the log-rank test. Uni-/multivariate analyses were computed by Cox proportional hazards model. Spearman r correlation analysis was applied to calculate bivariate correlations among the study variables.

### Ethics declarations

All methods in this research were performed in accordance with the principles of the Declaration of Helsinki. The study was approved by the Ethics Committee of the Fourth Hospital of Hebei Medical University.

## Supplementary Information

Supplementary Information.

## Data Availability

The data generated and/or analyzed during the current study are available from the corresponding author on reasonable request.

## References

[1] Sung H (2021). Global cancer statistics 2020: GLOBOCAN estimates of incidence and mortality worldwide for 36 cancers in 185 countries. CA Cancer J. Clin..

[2] Kamangar F, Dores GM, Anderson WF (2006). Patterns of cancer incidence, mortality, and prevalence across five continents: Defining priorities to reduce cancer disparities in different geographic regions of the world. J. Clin. Oncol..

[3] Guohong Z (2010). Genetic heterogeneity of oesophageal cancer in high-incidence areas of southern and northern China. PLoS ONE.

[4] Ishihara R (2010). Factors predictive of tumor recurrence and survival after initial complete response of esophageal squamous cell carcinoma to definitive chemoradiotherapy. Int. J. Radiat. Oncol. Biol. Phys..

[5] Pennathur A, Gibson MK, Jobe BA, Luketich JD (2013). Oesophageal carcinoma. Lancet.

[6] Buckley AM, Lynam-Lennon N, O'Neill H, O'Sullivan J (2020). Targeting hallmarks of cancer to enhance radiosensitivity in gastrointestinal cancers. Nat. Rev. Gastroenterol. Hepatol..

[7] Chen GZ (2017). The mechanisms of radioresistance in esophageal squamous cell carcinoma and current strategies in radiosensitivity. J. Thorac. Dis..

[8] Xu Y (2022). A phase III multicenter randomized clinical trial of 60 Gy versus 50 Gy radiation dose in concurrent chemoradiotherapy for inoperable esophageal squamous cell carcinoma. Clin. Cancer Res..

[9] Yang Y (2020). Impact of radiation dose on survival for esophageal squamous cell carcinoma treated with neoadjuvant chemoradiotherapy. Front. Oncol..

[10] Myatt SS, Lam EW (2007). The emerging roles of forkhead box (Fox) proteins in cancer. Nat. Rev. Cancer..

[11] Shi X (2012). Foxk1 promotes cell proliferation and represses myogenic differentiation by regulating Foxo4 and Mef2. J. Cell Sci..

[12] Grant GD (2012). Live-cell monitoring of periodic gene expression in synchronous human cells identifies Forkhead genes involved in cell cycle control. Mol. Biol. Cell..

[13] Sukonina V (2019). FOXK1 and FOXK2 regulate aerobic glycolysis. Nature.

[14] Bowman CJ, Ayer DE, Dynlacht BD (2014). Foxk proteins repress the initiation of starvation-induced atrophy and autophagy programs. Nat. Cell Biol..

[15] Hawke TJ, Jiang N, Garry DJ (2003). Absence of p21CIP rescues myogenic progenitor cell proliferative and regenerative capacity in Foxk1 null mice. J. Biol. Chem..

[16] Tang M (2020). FOXK1 participates in DNA damage response by controlling 53BP1 function. Cell. Rep..

[17] Guo X, Wang Y (2020). LncRNA TMPO-AS1 promotes hepatocellular carcinoma cell proliferation, migration and invasion through sponging miR-329-3p to stimulate FOXK1-mediated AKT/mTOR signaling pathway. Cancer Med..

[18] Peng Y (2016). Direct regulation of FOXK1 by C-jun promotes proliferation, invasion and metastasis in gastric cancer cells. Cell Death Dis..

[19] Wu M (2016). FOXK1 interaction with FHL2 promotes proliferation, invasion and metastasis in colorectal cancer. Oncogenesis..

[20] Wencong M (2020). FOXK1 promotes proliferation and metastasis of gallbladder cancer by activating AKT/mTOR signaling pathway. Front. Oncol..

[21] Chen D (2017). FOXK1 plays an oncogenic role in the development of esophageal cancer. Biochem. Biophys. Res. Commun..

[22] Pawlik TM, Keyomarsi K (2004). Role of cell cycle in mediating sensitivity to radiotherapy. Int. J. Radiat. Oncol. Biol. Phys..

[23] Biau J, Chautard E, Verrelle P, Dutreix M (2019). Altering DNA repair to improve radiation therapy: Specific and multiple pathway targeting. Front. Oncol..

[24] Castro-Mondragon JA (2022). JASPAR 2022: The 9th release of the open-access database of transcription factor binding profiles. Nucleic Acids Res..

[25] Huang E (2020). CircRNA hsa_circ_0004771 promotes esophageal squamous cell cancer progression via miR-339-5p/CDC25A axis. Epigenomics.

[26] Luo A (2019). Exosome-derived miR-339-5p mediates radiosensitivity by targeting Cdc25A in locally advanced esophageal squamous cell carcinoma. Oncogene.

[27] Fassl A, Geng Y, Sicinski P (2022). CDK4 and CDK6 kinases: From basic science to cancer therapy. Science.

[28] Yang Y (2020). CDK4/6 inhibitors: A novel strategy for tumor radiosensitization. J. Exp. Clin. Cancer Res..

[29] Benayoun BA, Caburet S, Veitia RA (2011). Forkhead transcription factors: Key players in health and disease. Trends Genet..

[30] Xie R (2017). RUFY3 interaction with FOXK1 promotes invasion and metastasis in colorectal cancer. Sci. Rep..

[31] Meng F (2021). SNHG1 knockdown upregulates miR-376a and downregulates FOXK1/Snail axis to prevent tumor growth and metastasis in HCC. Mol. Ther. Oncolytics..

[32] Rogakou EP, Pilch DR, Orr AH, Ivanova VS, Bonner WM (1998). DNA double-stranded breaks induce histone H2AX phosphorylation on serine 139. J. Biol. Chem..

[33] Celeste A (2002). Genomic instability in mice lacking histone H2AX. Science.

[34] Bassing CH (2002). Increased ionizing radiation sensitivity and genomic instability in the absence of histone H2AX. Proc. Natl. Acad. Sci. USA..

[35] Kao J (2006). gamma-H2AX as a therapeutic target for improving the efficacy of radiation therapy. Curr. Cancer Drug Targets..

[36] McIlwrath AJ, Vasey PA, Ross GM, Brown R (1994). Cell cycle arrests and radiosensitivity of human tumor cell lines: dependence on wild-type p53 for radiosensitivity. Can. Res..

[37] Baro M, Lopez Sambrooks C, Quijano A, Saltzman WM, Contessa J (2019). Oligosaccharyltransferase inhibition reduces receptor tyrosine kinase activation and enhances glioma radiosensitivity. Clin. Cancer Res..

[38] Hoffmann I, Draetta G, Karsenti E (1994). Activation of the phosphatase activity of human cdc25A by a cdk2-cyclin E dependent phosphorylation at the G1/S transition. EMBO J..

[39] Cangi MG (2000). Role of the Cdc25A phosphatase in human breast cancer. J. Clin. Invest..

[40] Guo P (2022). BPTF inhibition antagonizes colorectal cancer progression by transcriptionally inactivating Cdc25A. Redox Biol..

[41] Ding FN (2019). miR-122-5p modulates the radiosensitivity of cervical cancer cells by regulating cell division cycle 25A (CDC25A). FEBS Open Bio.

[42] Li H (2019). MiR-365 enhances the radiosensitivity of non-small cell lung cancer cells through targeting CDC25A. Biochem. Biophys. Res. Commun..

[43] Lim S, Kaldis P (2013). Cdks, cyclins and CKIs: Roles beyond cell cycle regulation. Development.

[44] Gao X, Leone GW, Wang H (2020). Cyclin D-CDK4/6 functions in cancer. Adv. Cancer Res..

[45] Patnaik A (2016). Efficacy and safety of abemaciclib, an inhibitor of CDK4 and CDK6, for patients with breast cancer, non-small cell lung cancer, and other solid tumors. Cancer Discov..

[46] Kim ES (2018). Abemaciclib in combination with single-agent options in patients with stage IV non-small cell lung cancer: A phase Ib study. Clin. Cancer Res..

[47] O'Leary B, Finn RS, Turner NC (2016). Treating cancer with selective CDK4/6 inhibitors. Nat. Rev. Clin. Oncol..

[48] Dall’Acqua A (2021). Inhibition of CDK4/6 as therapeutic approach for ovarian cancer patients: Current evidences and future perspectives. Cancers.

[49] Shimura T (2011). Targeting the AKT/GSK3beta/cyclin D1/Cdk4 survival signaling pathway for eradication of tumor radioresistance acquired by fractionated radiotherapy. Int. J. Radiat. Oncol. Biol. Phys..

[50] Deng X (2013). miR-124 radiosensitizes human glioma cells by targeting CDK4. J. Neurooncol..

[51] Hagen KR (2013). Silencing CDK4 radiosensitizes breast cancer cells by promoting apoptosis. Cell Div..

[52] Naz S (2018). Abemaciclib, a selective CDK4/6 inhibitor, enhances the radiosensitivity of non-small cell lung cancer in vitro and in vivo. Clin. Cancer Res..

[53] Hashizume R (2016). Inhibition of DNA damage repair by the CDK4/6 inhibitor palbociclib delays irradiated intracranial atypical teratoid rhabdoid tumor and glioblastoma xenograft regrowth. Neuro Oncol..

