# Supplementary material for: FOXK1 regulates malignant progression and radiosensitivity through direct transcriptional activation of CDC25A and CDK4 in esophageal squamous cell carcinoma
Source: Sci Rep. 2023 May 12;13:7737. doi: 10.1038/s41598-023-34979-y (PMC10182098; doi:10.1038/s41598-023-34979-y)
Supplement: Supplementary file 1 — Supplementary Information. [file 41598_2023_34979_MOESM1_ESM.pdf]

FOXK1 regulates malignant progression and radiosensitivity through direct transcriptional activation of CDC25A and CDK4 in esophageal squamous cell carcinoma

Xiaoxu Li, Juntao Lu, Lei Liu, Fei Li, Tongxin Xu, Liying Chen, Zhaoyang Yan, Yan Li, Wei Guo

Supplementary Tables

Supplementary Table 1

The correlation between the expression level of FOXK1 and the clinicopathological characteristics of ESCC patients.

Characteristics	N	FOXK1 expression		χ^2	*P* value
		Low n (%)	High n (%)		
Age (years)				0.095	0.758
< 60	33	10 (29.4)	23 (32.4)		
≥ 60	72	24 (70.6)	48 (67.6)		
Gender				0.748	0.387
Male	68	24 (70.6)	44 (62.0)		
Female	37	10 (29.4)	27 (38.0)		
Pathological differentiation				0.609	0.435
Well/Moderate	49	14 (41.2)	35 (49.3)		
Poor	56	20 (58.8)	36 (50.7)		
TNM stage				10.171	0.017*
I	11	8 (23.5)	3 (4.2)		
II	33	11 (32.4)	22 (31.0)		
III	48	11 (32.4)	37 (52.1)		
IV	13	4 (11.8)	9 (12.7)		
Depth of invasion				8.574	0.036*
T1	13	8 (23.5)	5 (7.0)		
T2	30	12 (35.3)	18 (25.4)		
T3	48	11 (32.4)	37 (52.1)		
T4	14	3 (8.8)	11 (15.5)		
LN metastasis				5.375	0.020*
N0	39	18 (52.9)	21 (29.6)		
N1/2/3	66	16 (47.1)	50 (70.4)		

Distant metastasis				0.293	0.588
M0	95	30 (88.2)	65 (91.5)		
M1	10	4 (11.8)	6 (8.5)		
Family history of UGIC				0.332	0.565
Negative	83	28 (82.4)	55 (77.5)		
Positive	22	6 (17.6)	16 (22.5)		

* *P* < 0.05.

Supplementary Table 2

Univariate and multivariate Cox regression analysis of clinicopathological characteristics of 105 ESCC patients

Variables	Univariate analysis		Multivariate analysis	
	HR (95 % CI)	*P*	HR (95 % CI)	*P*
Age (< 60 vs. ≥ 60)	0.815 (0.507-1.311)	0.400	1.062 (0.619-1.828)	0.828
Gender (male vs. female)	0.968 (0.612-1.532)	0.890	0.903 (0.547-1.490)	0.689
Pathological differentiation (well/moderate vs. poor)	1.090 (0.703-1.691)	0.699	0.961 (0.603-1.530)	0.866
TNM stage (I/II vs. III/IV)	2.398 (1.521-3.782)	0.000**	0.886 (0.370-2.122)	0.786
Depth of invasion (T1/2 vs. T3/4)	3.535 (2.159-5.787)	0.000**	3.566 (1.975-6.438)	0.000**
LN metastasis (N0 vs. N1/2/3)	1.933 (1.215-3.076)	0.005**	2.423 (1.020-5.758)	0.045*
Distant metastasis (M0 vs. M1)	6.978 (3.291-14.795)	0.000**	6.647 (2.756-16.036)	0.000**
Family history of UGIC (negative vs. positive)	1.323 (0.782-2.238)	0.296	0.935 (0.534-1.637)	0.815
FOXK1 (low vs. high)	2.224 (1.369-3.613)	0.001**	2.246 (1.305-3.866)	0.003**

* *P* < 0.05; ** *P* < 0.01.

Supplementary Table 3

Radiation biological parameters in cell survival curves

	D0	N	Dq	SF2
**KYSE170**				
pcDNA3.1-NC	1.74±0.02	2.00±0.08	1.20±0.06	0.53±0.01
pcDNA3.1-FOXK1	2.46±0.05*	2.25±0.03*	2.00±0.07*	0.70±0.01*
**TE1**				

pcDNA3.1-NC	1.84±0.05	2.40±0.07	1.61±0.03	0.62±0.01
pcDNA3.1-FOXK1	2.61±0.06*	2.81±0.05*	2.70±0.12*	0.77±0.01*

Data are the mean of three independent experiments ± SEM. A comparison with the NC group is symbolized by the asterisk, * $P < 0.05$.

Supplementary Table 4

Primer sequences used for qPCR assays

Gene	Primer sequences (5'–3')
GAPDH	Forward: GAAGGTGAAGGTCGGAGTC
	Reverse: GAAGATGGTGATGGGATTTC
FOXK1	Forward: AGCAGTGTACCTTCCGGTTTC
	Reverse: GTGGATCTTCAGAGGGGAGATC
CCND3	Forward: AAACTTGGCTGAGCAGAGCAC
	Reverse: TGCAGTGGCGAAGTGTTTAC
CCNE2	Forward: TCAAGACGAAGTAGCCGTTTAC
	Reverse: TGACATCCTGGGTAGTTTTCCTC
CDC7	Forward: TGGGGATCTCTTGGAGACGG
	Reverse: AAGACGCCTCCATCACAAGC
CDC20	Forward: GCACAGTTCGCGTTCGAGA
	Reverse: CTGGATTTGCCAGGAGTTCGG
CDC23	Forward: CATGGCTGCAATAGCAAGAAAG
	Reverse: CGCCTCATTTTTCACTTGTCCT
CDC25A	Forward: TTCCTCTTTTTACACCCCAGTCA
	Reverse: TCGGTTGTCAAGGTTTGTAGTTC
CDC25C	Forward: ATGACAATGGAAACTTGGTGGAC
	Reverse: GGAGCGATATAGGCCACTTCTG
CDK1	Forward: GGCTCTTGGAAATTGAGCGG
	Reverse: AGGAACCCCTTCCTCTTCACT
CDK4	Forward: ATGGCTACCTCTCGATATGAGC
	Reverse: CATTGGGGACTCTCACACTCT
FGF12	Forward: ATTGAACTTCCCAGCACTCG
	Reverse: GTAGTCGCTGTTTTCGTCCT
FGFR2	Forward: GGTGGCTGAAAAACGGGAAG
	Reverse: AGATGGGACCACACTTTCCATA
FGFR4	Forward: CCATAGGGACCCCTCGAATAG
	Reverse: CAGCGGAACTTGACGGTGT
NOTCH1	Forward: GGACGTCAGACTTGGCTCAG
	Reverse: ACATCTTGGGACGCATCTGG
AKT2	Forward: AGGCACGGGCTAAAGTGAC

	Reverse: CTGTGTGAGCGACTTCATCCT
BMP6	Forward: AGCGACACCACAAAGAGTTCA
	Reverse: GCTGATGCTCCTGTAAGACTTGA
BMP7	Forward: TGCAGCCTCCACTCTCTAGT
	Reverse: GAGGCTGGTTGGGTCAGAAA
ROCK1	Forward: AAGTGAGGTTAGGGCGAAATG
	Reverse: AAGGTAGTTGATTGCCAACGAA
TGFB1	Forward: TGATGTCACCGGAGTTGTGC
	Reverse: GTAGTGAACCCGTTGATGTCCA
TGFBR2	Forward: GTCCTGTGGACGCGTATCG
	Reverse: TGGAAACTTGACTGCACCGT
VEGFA	Forward: ATGCGGATCAAACCTCACCA
	Reverse: CACCAACGTACACGCTCCAG
PDGFRA	Forward: CACAAAACCTCTTTCCCGGC
	Reverse: CGCCTTCGCTTTTTGCTCTT
DVL2	Forward: GGCTTTGGTGGCTGAAGACT
	Reverse: GGACTGTGGGGGTTCCTAGA
DVL3	Forward: TCTGGGGACCAAAGCAATCC
	Reverse: TCCCTAGAAACGGAAGGGGA
FZD1	Forward: CGGTAAAATCTAAGCGCAGG
	Reverse: AGCTTTGTGTGGGTTGGAAG
FZD3	Forward: GTTCATGGGGCATATAGGTGG
	Reverse: GCTGCTGTCTGTTGGTCATAA
FZD6	Forward: CAGCGCCAAGAGCTTCAAAA
	Reverse: CTCCACCTTGCCGTCTGTTA

Supplementary Table 5

Primers used for si-RNA sequences

**Names**	**Sequence (5'–3')**
si-FOXK1-1	CCGGCACAACCUCUCUUUG
si-FOXK1-2	CCAUCAAGAUCCAGUUCAC
si-CDC25A-1	GGGCAGUGAUUAUGAGCAA
si-CDC25A-2	GGAAAAUGAAGCCUUUGAG
si-CDK4-1	UGCUGACUUUUAACCCACA
si-CDK4-2	CCAGAAUCUACAGCUACCA

Supplementary Table 6

Antibodies used for Western blot and ChIP assays

Antibody	Company	Catalogue number
β-Actin	ZenBioScience	380624
FOXK1	Abcam	ab85999
γH2AX	Abcam	ab81299
Cyclin D1	ZenBioScience	380999
CDK4	ZenBioScience	383886
P21	ZenBioScience	381102
Bcl-2	ZenBioScience	381702
Bax	ZenBioScience	380709
CDC25A	ZenBioScience	160979

Supplementary Table 7

Primers used for the construction of luciferase reporter and ChIP-PCR assays

Gene	Region on promoter	Forward primer (5'–3')	Reverse primer (5'–3')
**Primers for the construction of luciferase reporters**			
CDC25A	-1802/+76	CTAGCTAGCTTGTGGTATTATTTCAAGCACT	GTTAAGCTTGAAAACCAAGCCGACCTACACC
	-961/+76	CTAGCTAGCACTTATGGCCCTCAAAATCCGTCT	GTTAAGCTTGAAAACCAAGCCGACCTACACC
	-590/+76	CTAGCTAGCAGGGTCTGTGAGCCCTCCAGAGT	GTTAAGCTTGAAAACCAAGCCGACCTACACC
CDK4	-1328/+22	CTAGCTAGCTCCTCCTTTACTCCCATACCCC	CCCAAGCTTCCATAGACACAGGCCGCAAG
	-865/+22	CTAGCTAGCAACTTCTCTGCCAGATGGCCTC	CCCAAGCTTCCATAGACACAGGCCGCAAG
	-511/+22	CTAGCTAGCTGGTGGAGCGAAAAGGTGACA	CCCAAGCTTCCATAGACACAGGCCGCAAG
**Primers for ChIP-PCR**			
CDC25A	-974/-871	AATTAAATCATGCACTTATG	ATGTCACAGAGTTGTAAAG
CDK4	-758/-657	TATTAATCTGCCTTCCGTCAT	AGATAGTCGTACCACTACTTT

Supplementary figures

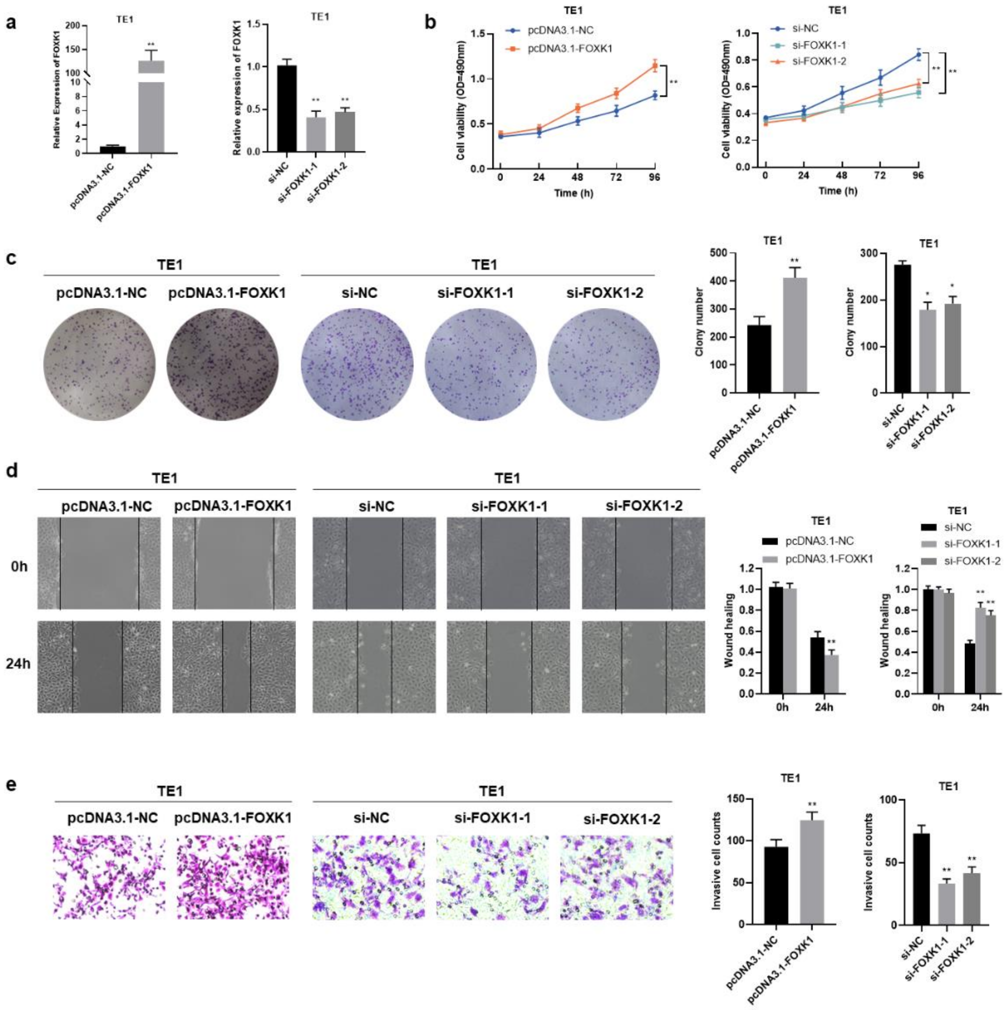

Supplementary figure 1. FOXK1 promotes proliferation, migration, and invasion of TE1 cells. (**a**) The transfection efficiency of overexpression of FOXK1 and knockdown of FOXK1 in TE1 cells, respectively, detected using qRT-PCR assay. (**b, c**) The effects of upregulation and downregulation of FOXK1 on proliferation of TE1 cells were examined using MTS (**b**) and colony formation (**c**) assays. (**d, e**) Wound healing (**d**) and transwell (**e**) assays were performed to examine the influences of overexpression and knockdown of FOXK1 on the migration and invasion of TE1 cells. Values represent the Mean ± SD for three

independent experiments. Statistical significance was determined as * $P < 0.05$ and ** $P < 0.01$.

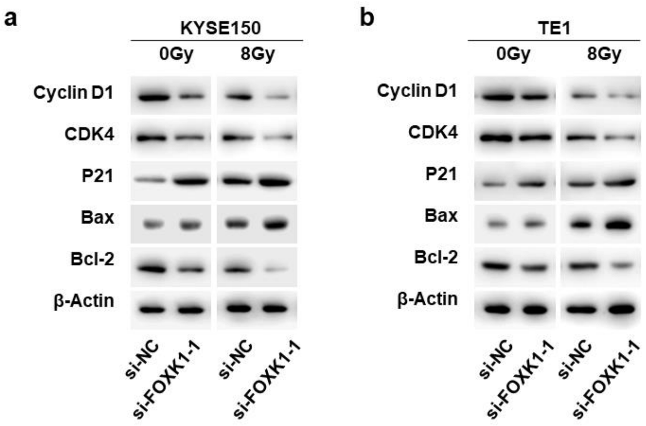

Supplementary figure 2. Effect of knockdown of FOXK1 on irradiated ESCC cell cycle and apoptosis associated proteins. **(a, b)** Western blot assays were performed to examine the protein expression levels of cyclin D1, CDK4, P21, Bax, and Bcl-2 in FOXK1-deficient irradiated and non-irradiated KYSE150 **(a)** and TE1 cells **(b)**. Original western blots were presented in Supplementary Fig. 6h, with blots cut prior to hybridization with antibodies.

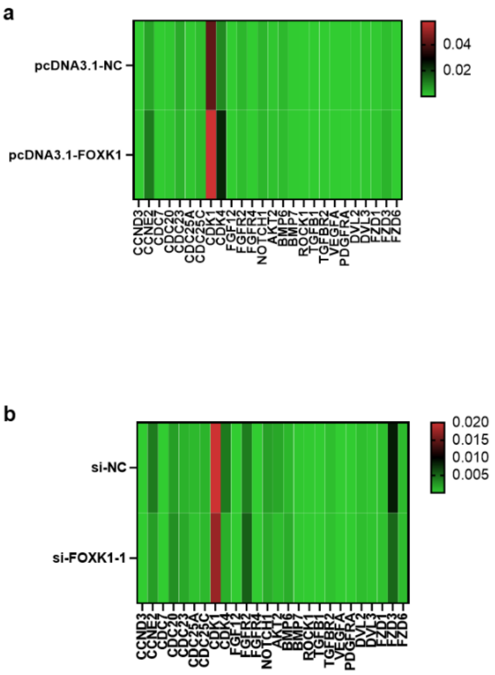

Supplementary figure 3. Screening for potential downstream target genes of FOXK1. (**a**) The expression levels of several genes in FOXK1 overexpressing KYSE170 cells were determined by qRT-PCR assays. (**b**) The mRNA expression levels of several genes in FOXK1 knockdown KYSE150 cells, detected by qRT-PCR assay.

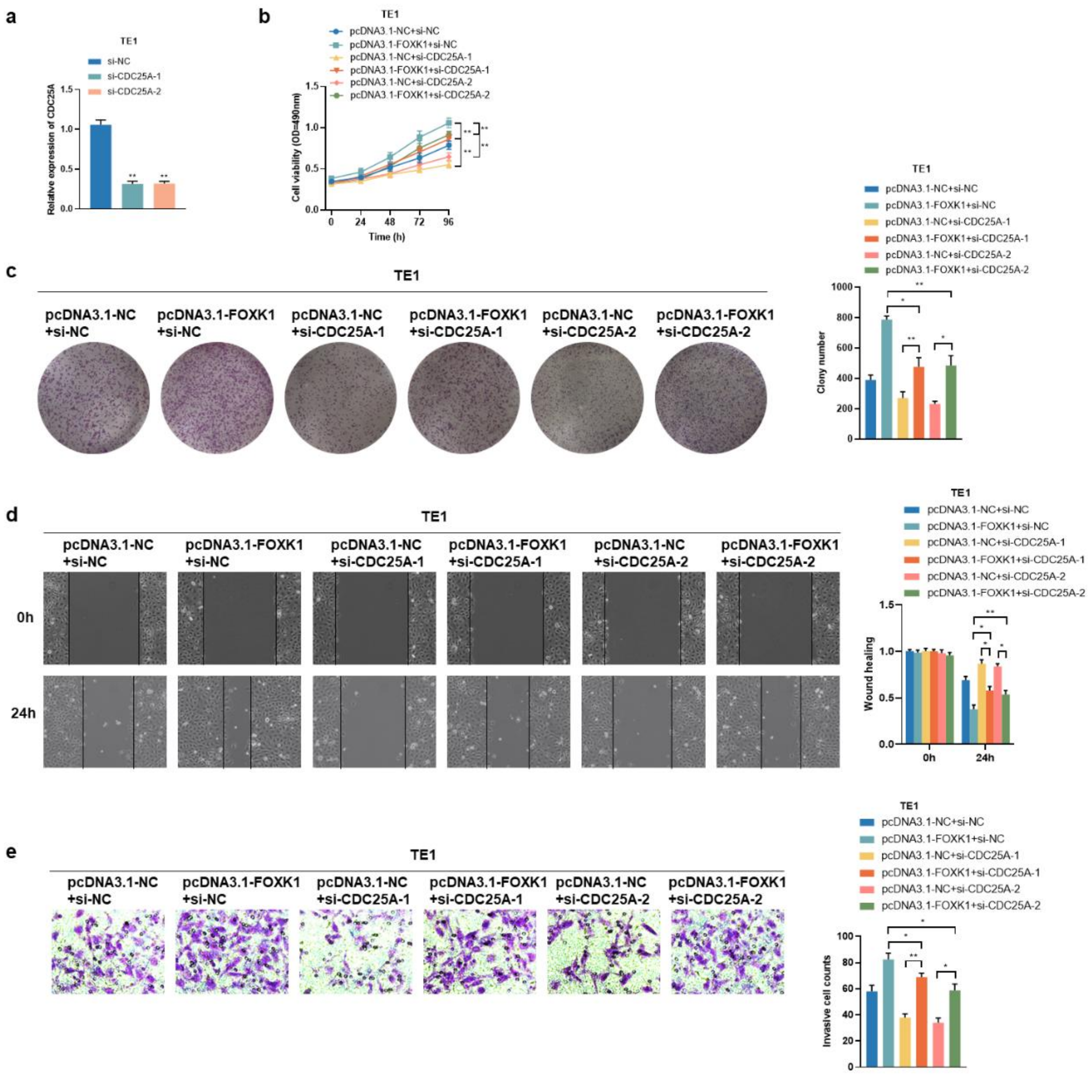

Supplementary figure 4. FOXK1 enhances proliferation, migration and invasion of TE1 cells through upregulation of CDC25A. (**a**) The transfection efficiency of CDC25A knockdown in TE1 cells. (**b, c**) MTS (**b**) and colony formation (**c**) assays were applied to verify the effect of downregulation of CDC25A on the proliferative ability of FOXK1 overexpressing TE1 cells. (**d, e**) The effect of CDC25A depletion on the migratory and invasive capacity of FOXK1 upregulating TE1 cells was confirmed by wound healing (**d**) and transwell (**e**) assays. Data presented are the mean ± SD from at least three independent experiments. * $P < 0.05$, ** $P < 0.01$.

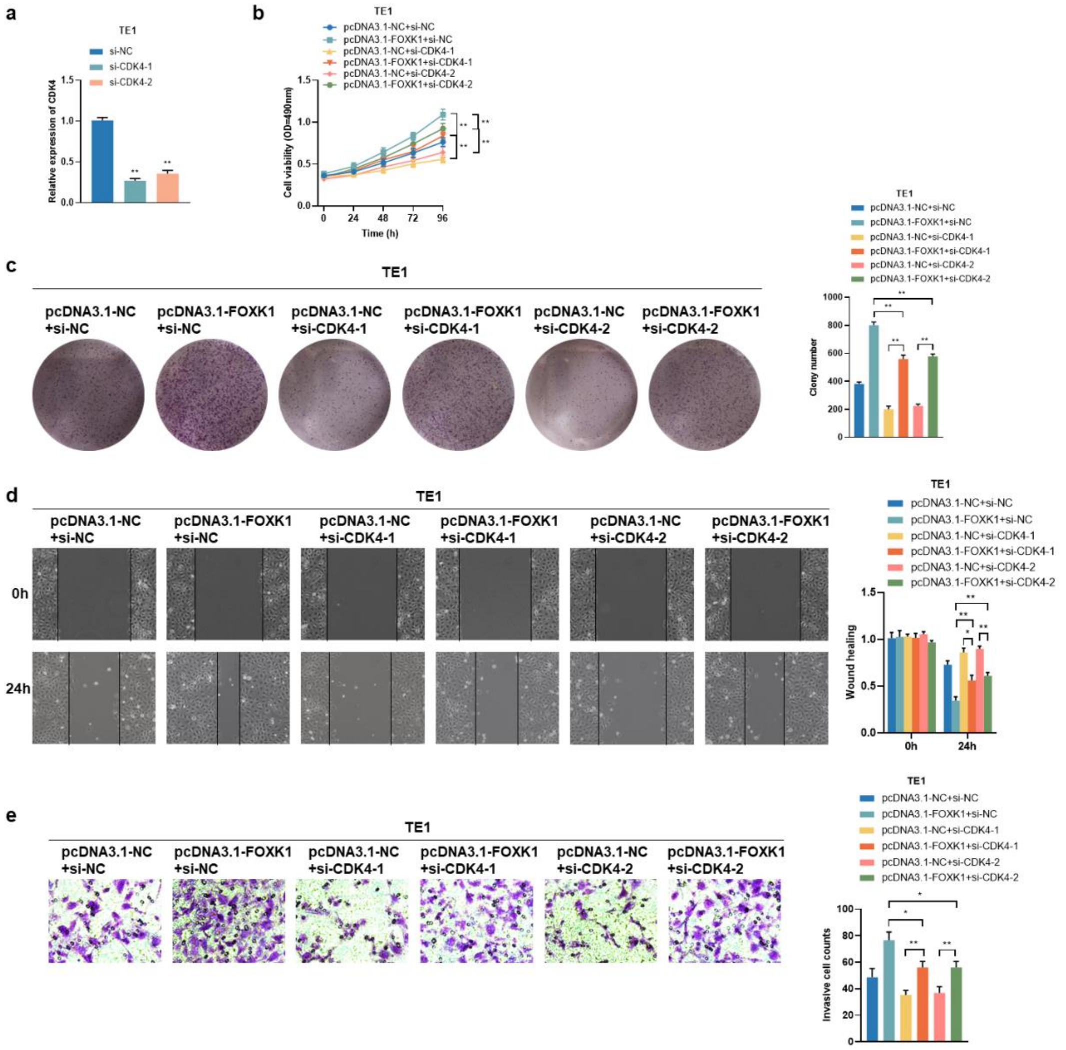

Supplementary figure 5. FOXK1 enhances the malignant processes of TE1 cells through upregulation of CDK4. (**a**) The transfection efficiency of CDK4 knockdown in TE1 cells was determined by qRT-PCR assays. (**b, c**) The effect of knockdown of CDK4 on the proliferation of FOXK1 overexpressing TE1 cells was examined using MTS (**b**) and colony formation (**c**) assays. (**d, e**) The role of reduction of CDK4 on the migration and invasion ability of FOXK1 upregulating TE1 cells was detected using wound healing (**d**) and transwell (**e**) assays. Data presented are the mean ± SD from at least three independent experiments. * $P < 0.05$, ** $P < 0.01$.

a

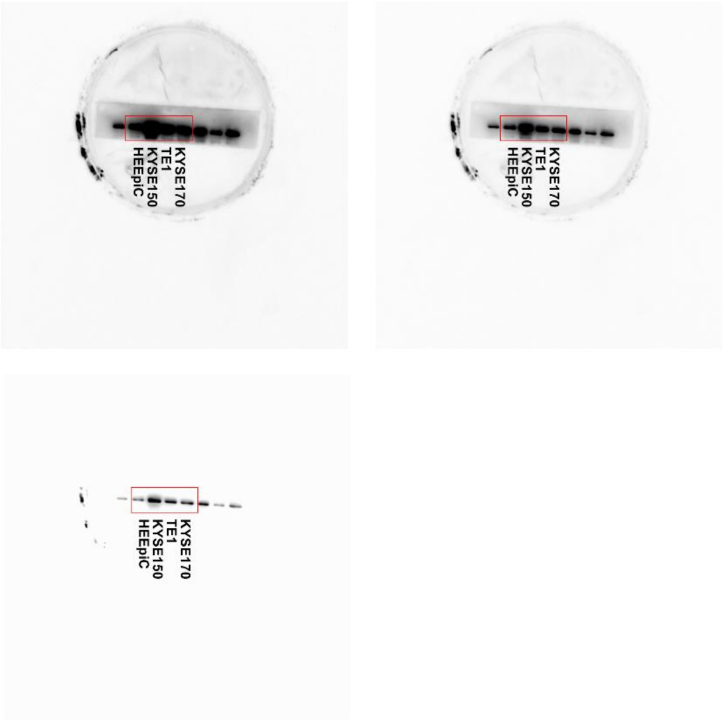

FOXK1 (protein bands of FOXK1 in different cell lines in Figure 1a).

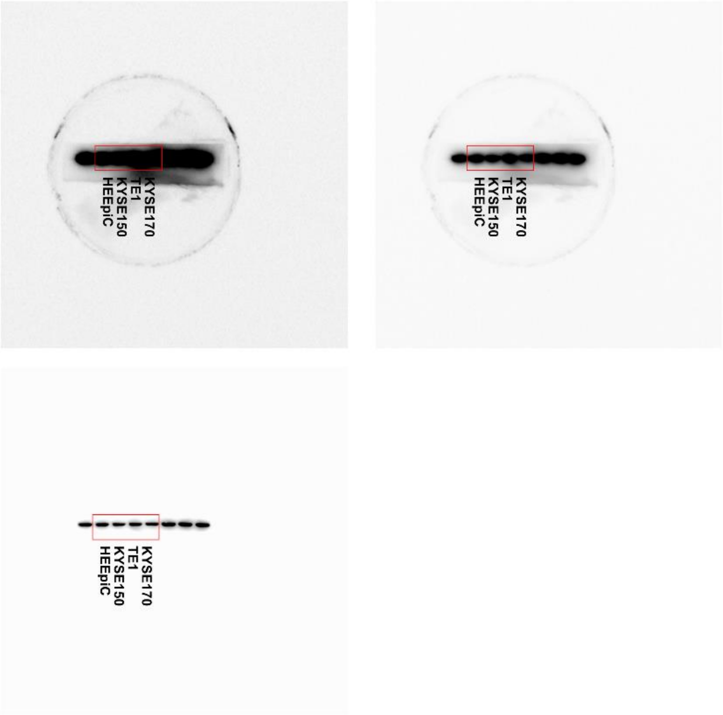

β-Actin (protein bands of β-Actin in different cell lines in Figure 1a).

b

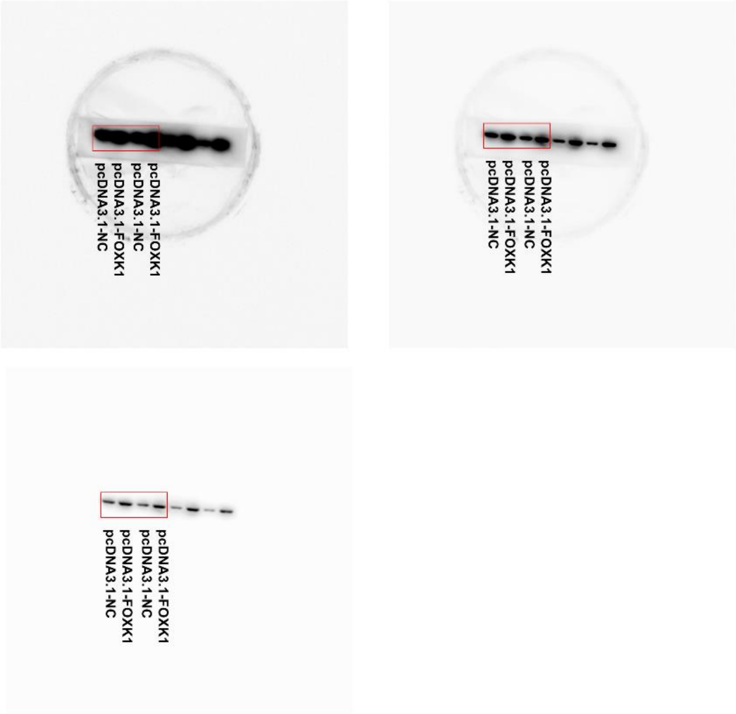

FOXK1 (protein bands of FOXK1 in KYSE170 and TE1 cells after performing different transfections in Figure 3a).

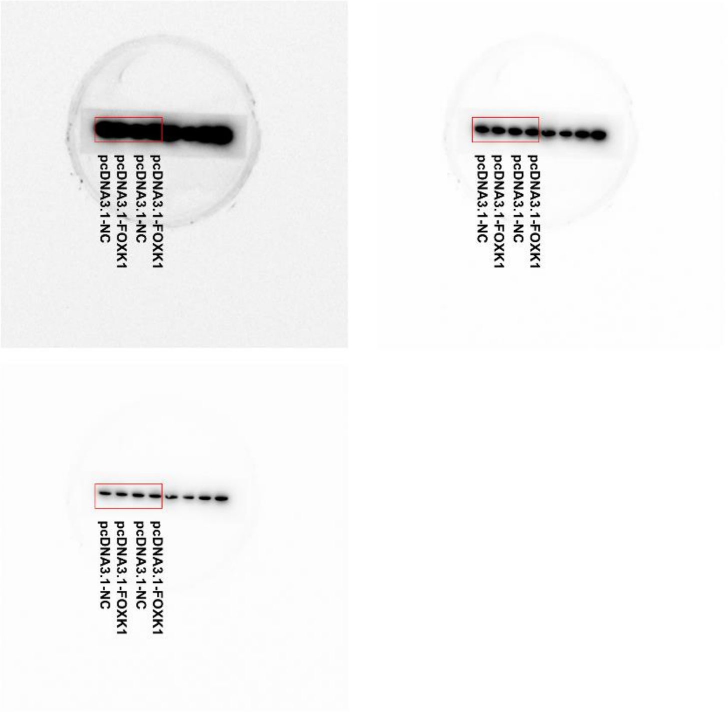

β-Actin (protein bands of β-Actin in KYSE170 and TE1 cells after performing different transfections in Figure 3a).

c

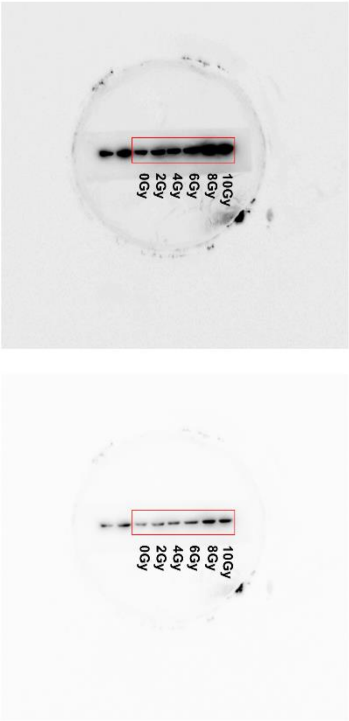

FOXK1 (protein bands of FOXK1 in KYSE170 cells under graded radiation doses in Figure 3d).

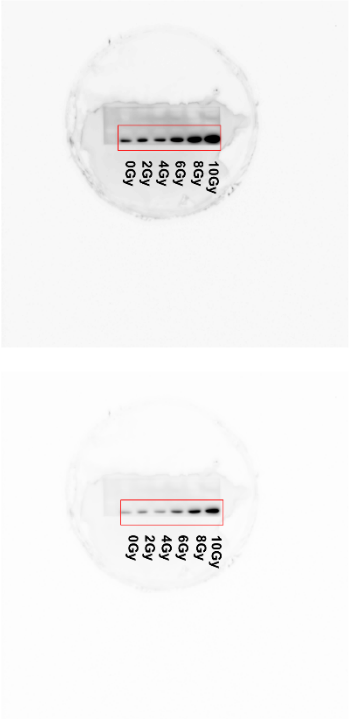

γH2AX (protein bands of γH2AX in KYSE170 cells under graded radiation doses in Figure 3d).

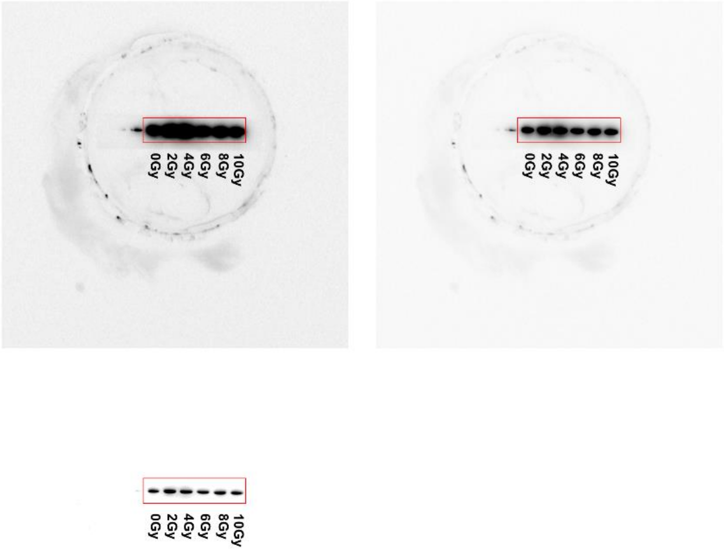

β-Actin (protein bands of β-Actin in KYSE170 cells under graded radiation doses in Figure 3d).

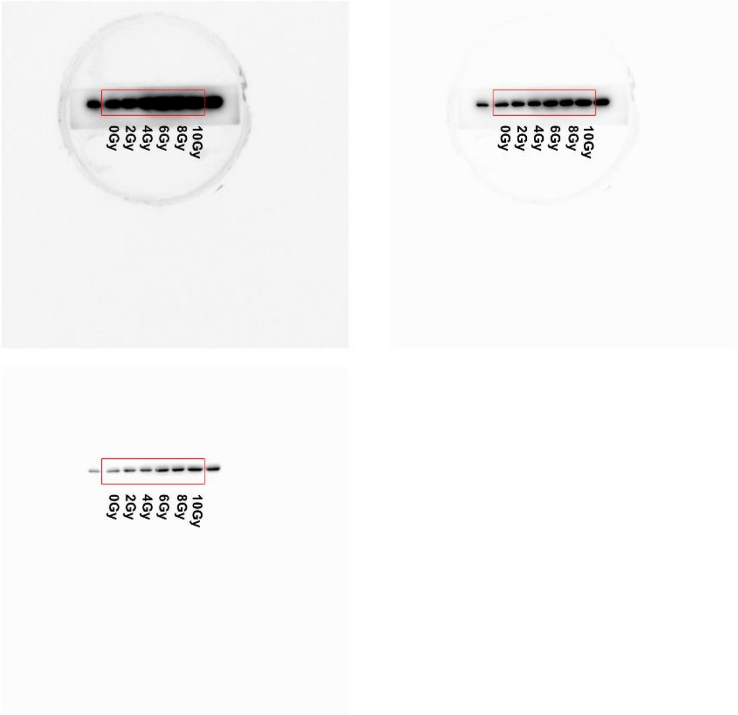

FOXK1 (protein bands of FOXK1 in TE1 cells under graded radiation doses in Figure 3d).

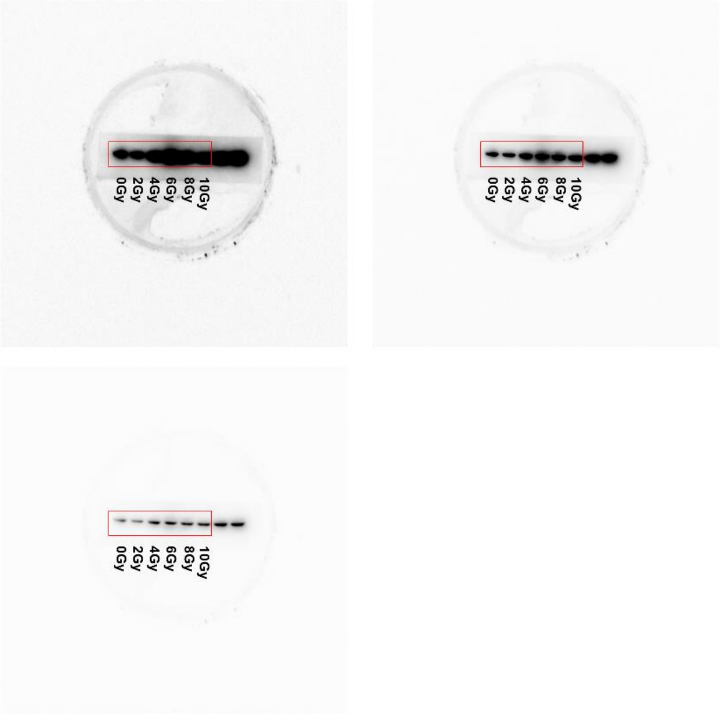

γH2AX (protein bands of γH2AX in TE1 cells under graded radiation doses in Figure 3d).

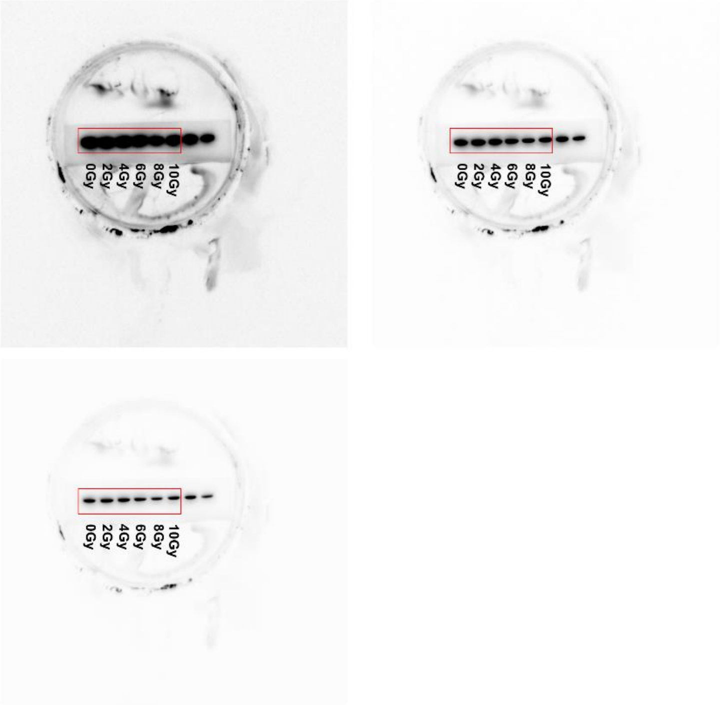

β-Actin (protein bands of β-Actin in TE1 cells under graded radiation doses in Figure 3d).

d

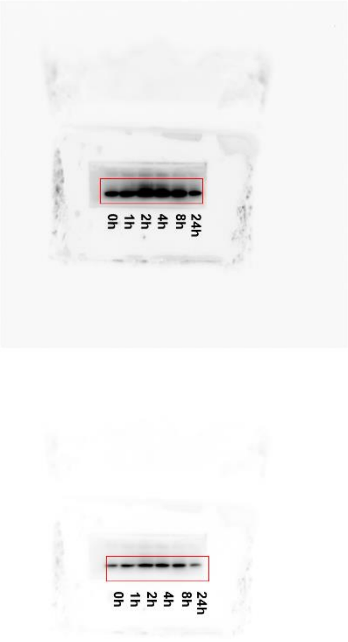

FOXK1 (protein bands of FOXK1 in KYSE170 cells at different time points after irradiation in Figure 3e).

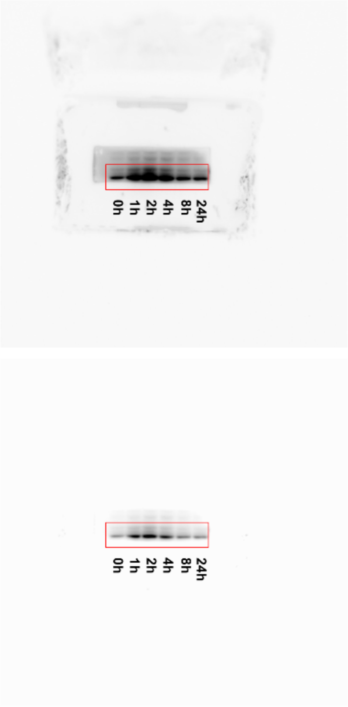

γH2AX (protein bands of γH2AX in KYSE170 cells at different time points after irradiation in Figure 3e).

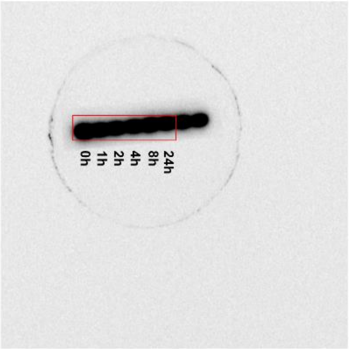

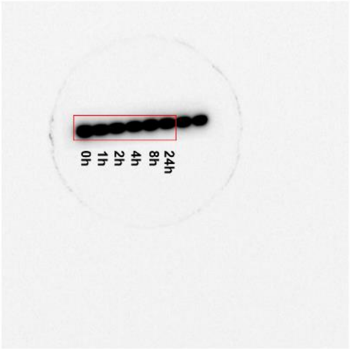

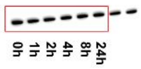

β-Actin (protein bands of β-Actin in KYSE170 cells at different time points after irradiation in Figure 3e).

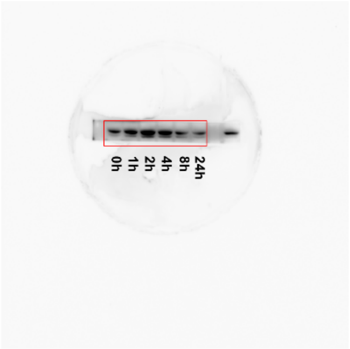

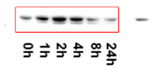

FOXK1 (protein bands of FOXK1 in TE1 cells at different time points after irradiation in Figure 3e).

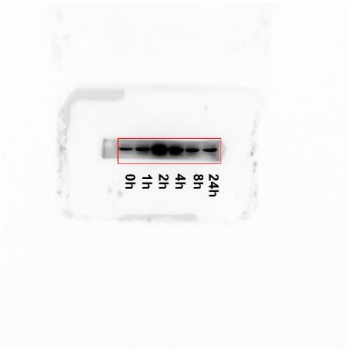

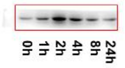

γH2AX (protein bands of γH2AX in TE1 cells at different time points after irradiation in Figure 3e).

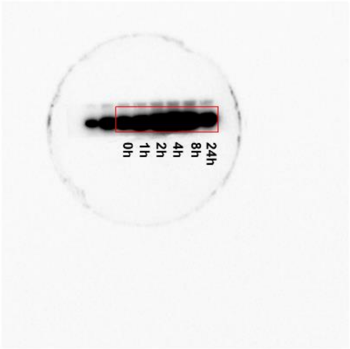

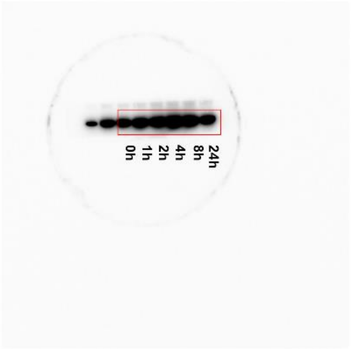

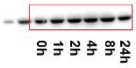

β-Actin (protein bands of β-Actin in TE1 cells at different time points after irradiation in Figure 3e).

e

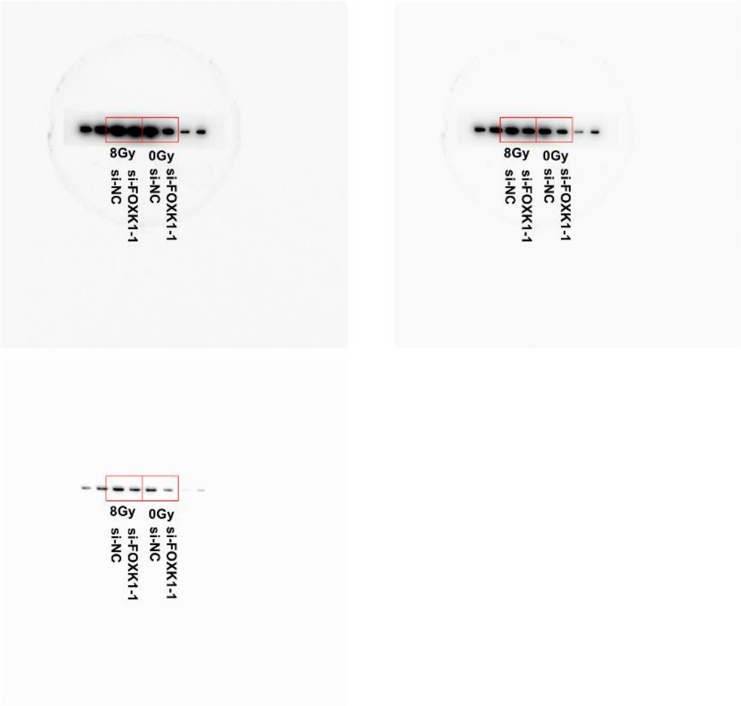

FOXK1 (protein bands of FOXK1 in irradiated and non-irradiated KYSE150 cells with different transfection treatments in Figure 3f).

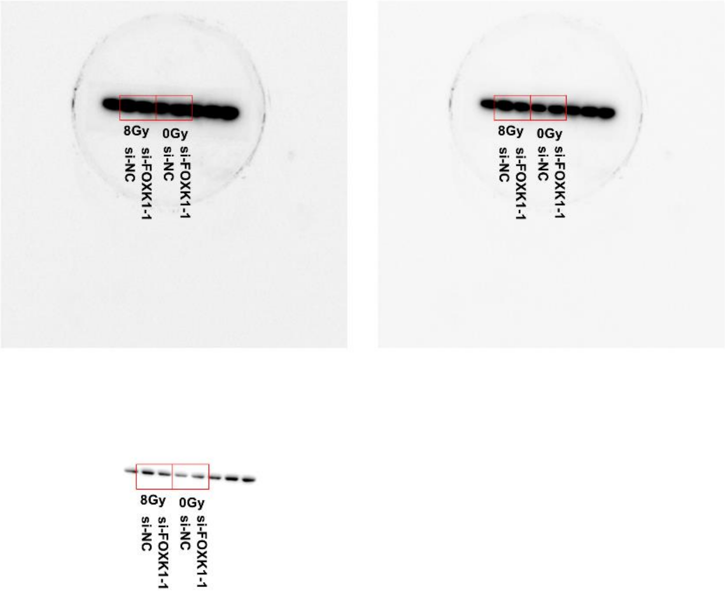

γH2AX (protein bands of γH2AX in irradiated and non-irradiated KYSE150 cells with different transfection treatments in Figure 3f).

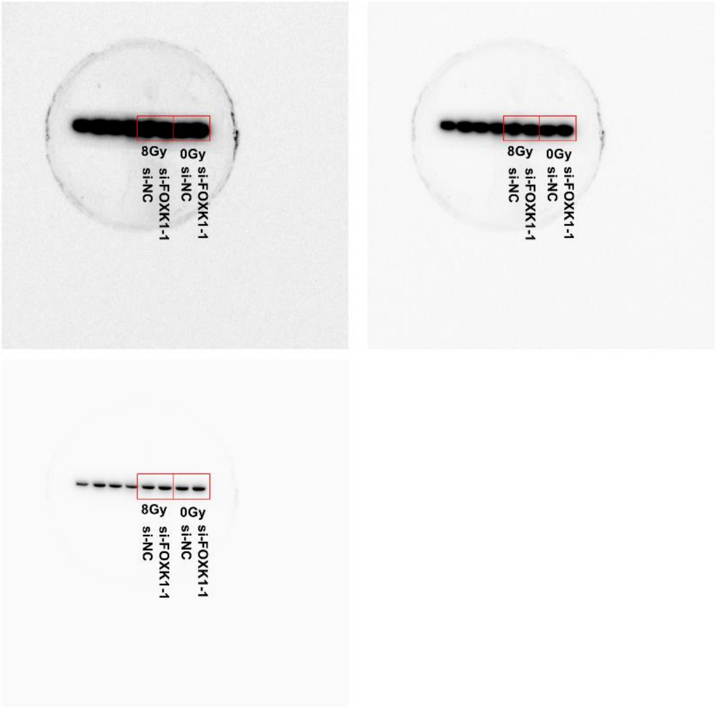

β-Actin (protein bands of β-Actin in irradiated and non-irradiated TE1 cells with different transfection treatments in Figure 3f).

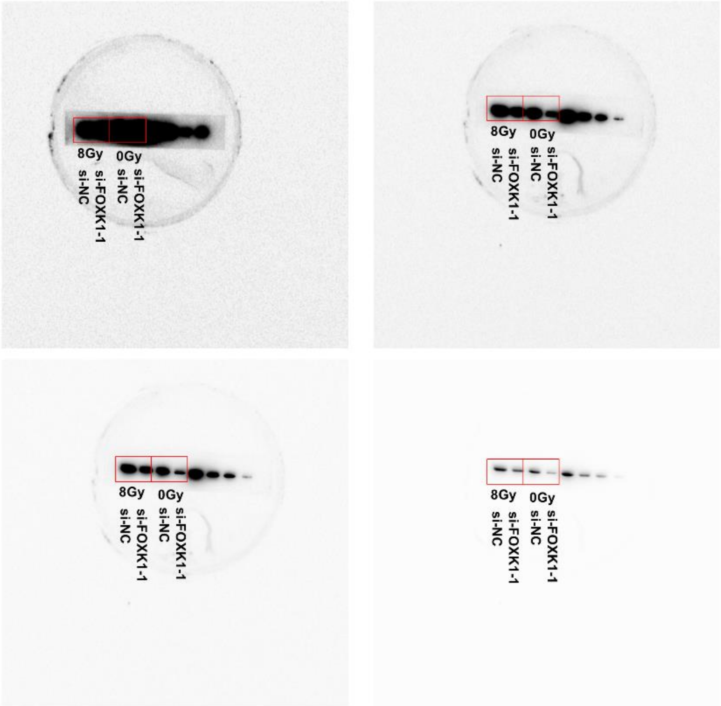

FOXK1 (protein bands of FOXK1 in irradiated and non-irradiated TE1 cells with different transfection treatments in Figure 3f).

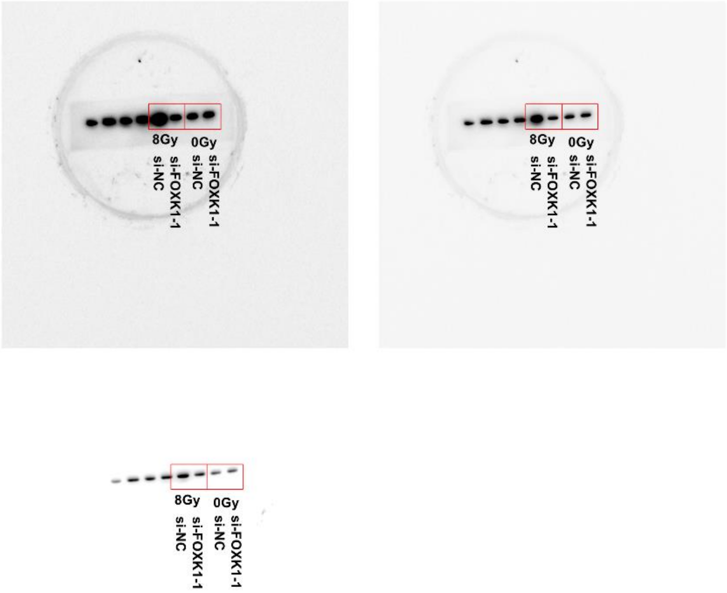

γH2AX (protein bands of γH2AX in irradiated and non-irradiated TE1 cells with different transfection treatments in Figure 3f).

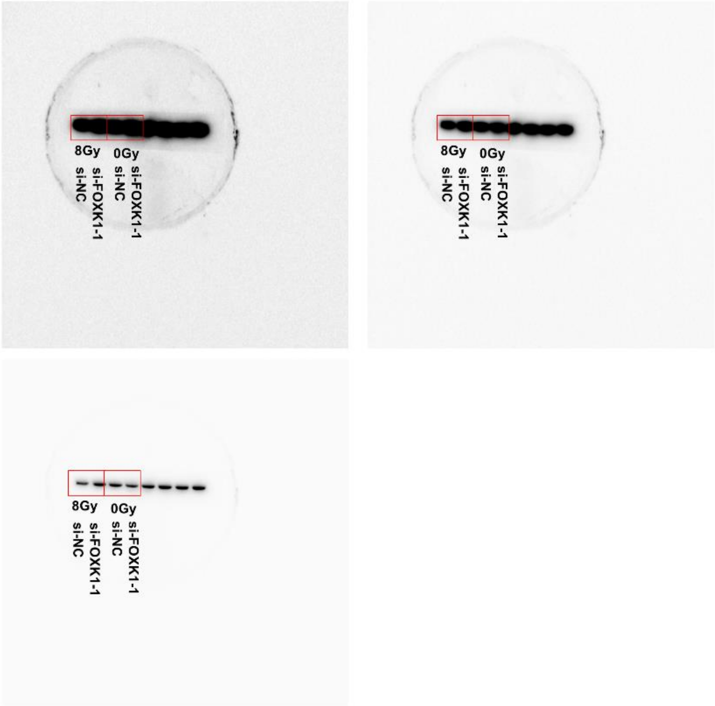

β-Actin (protein bands of β-Actin in irradiated and non-irradiated TE1 cells with different transfection treatments in Figure 3f).

f

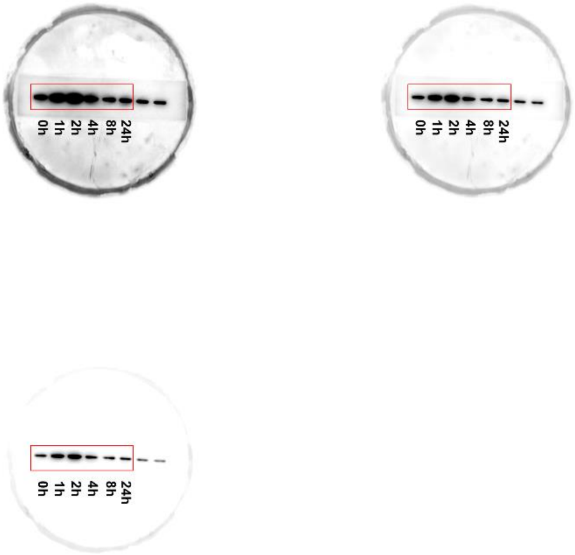

FOXK1 (protein bands of FOXK1 in si-NC group of KYSE150 cells at different time points after irradiation in Figure 3g).

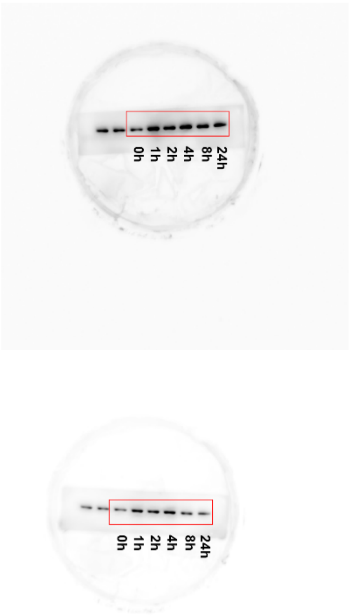

γH2AX (protein bands of γH2AX in si-NC group of KYSE150 cells at different time points after irradiation in Figure 3g).

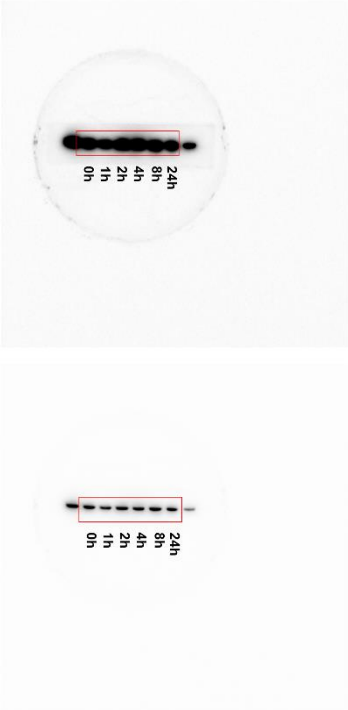

β-Actin (protein bands of β-Actin in si-NC group of KYSE150 cells at different time points after irradiation in Figure 3g).

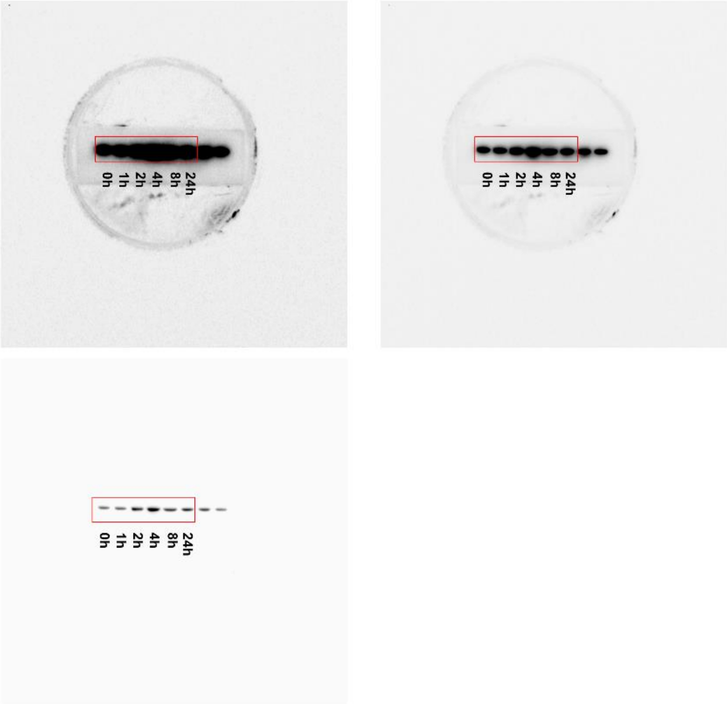

FOXK1 (protein bands of FOXK1 in si-FOXK1-1 group of KYSE150 cells at different time points after irradiation in Figure 3g).

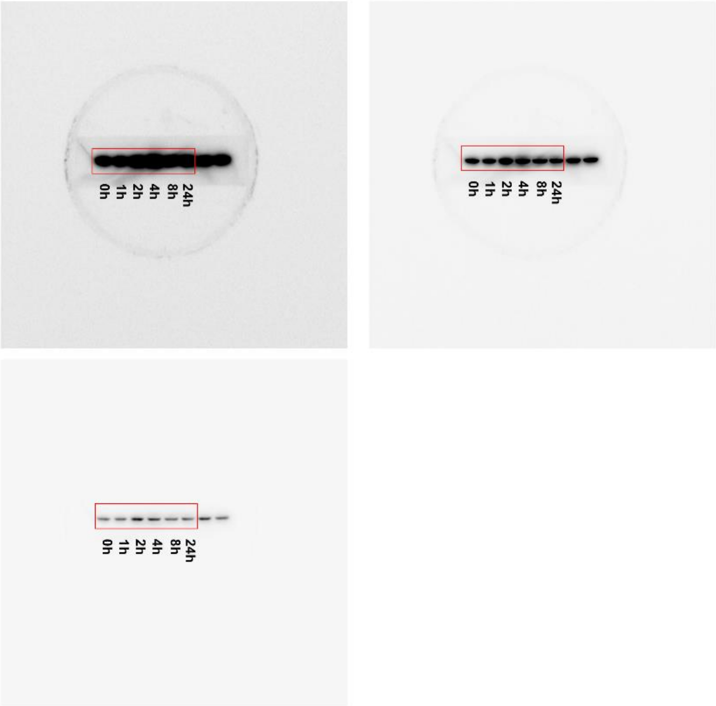

γH2AX (protein bands of γH2AX in si-FOXK1-1 group of KYSE150 cells at different time points after irradiation in Figure 3g).

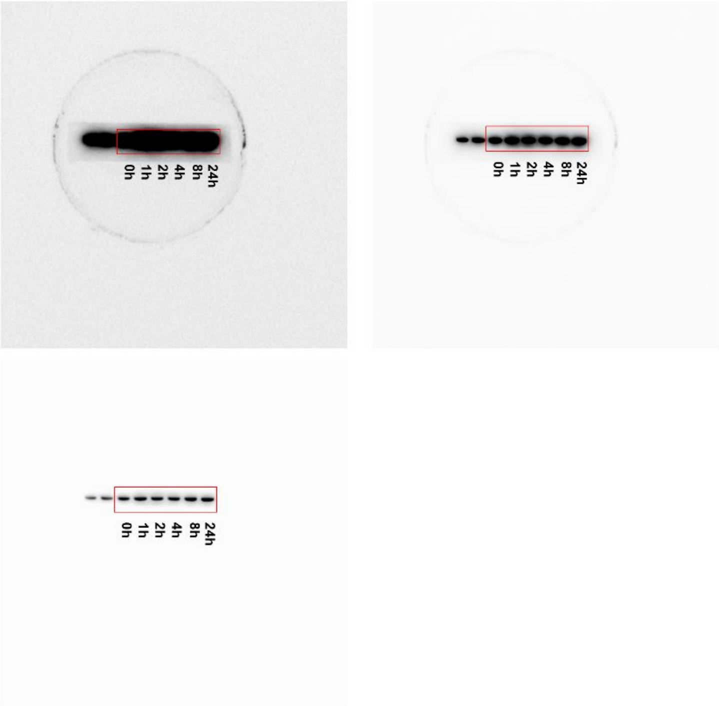

β-Actin (protein bands of β-Actin in si-FOXK1-1 group of KYSE150 cells at different time points after irradiation in Figure 3g).

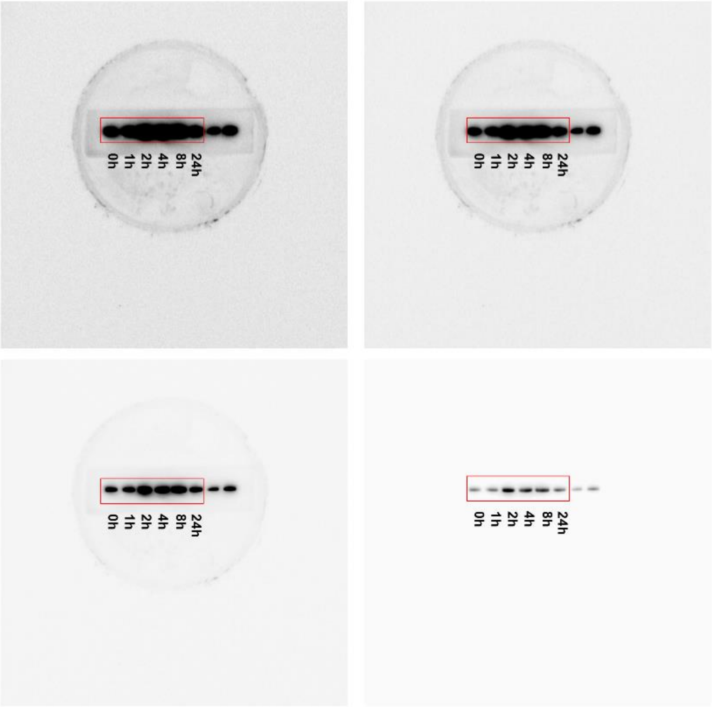

FOXK1 (protein bands of FOXK1 in si-FOXK1-2 group of KYSE150 cells at different time points after irradiation in Figure 3g).

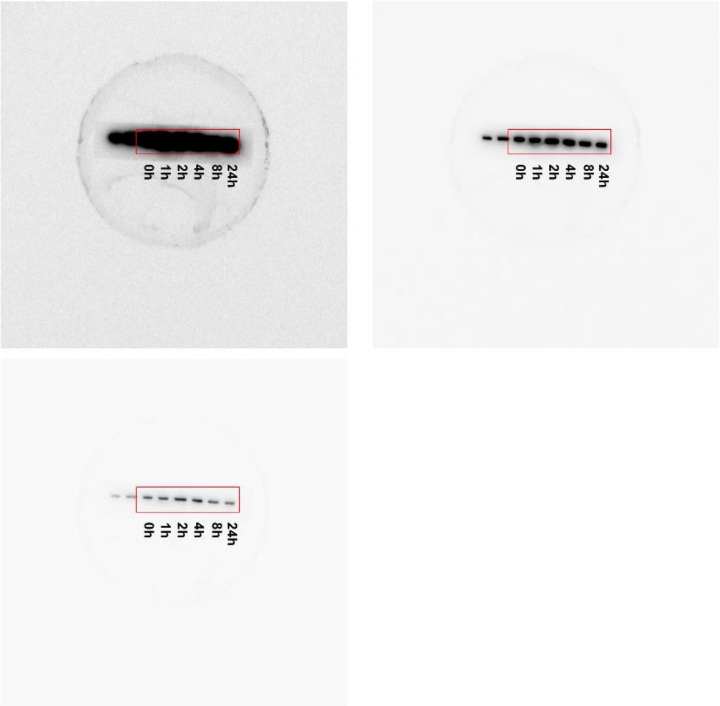

γH2AX (protein bands of γH2AX in si-FOXK1-2 group of KYSE150 cells at different time points after irradiation in Figure 3g).

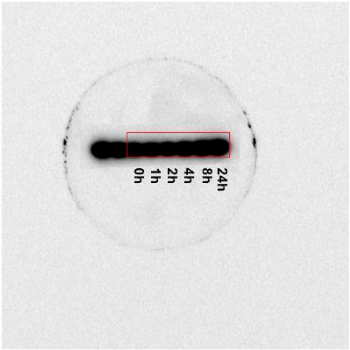

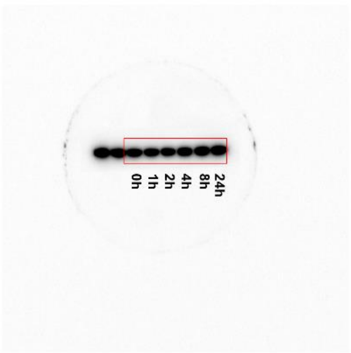

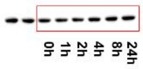

β-Actin (protein bands of β-Actin in si-FOXK1-2 group of KYSE150 cells at different time points after irradiation in Figure 3g).

g

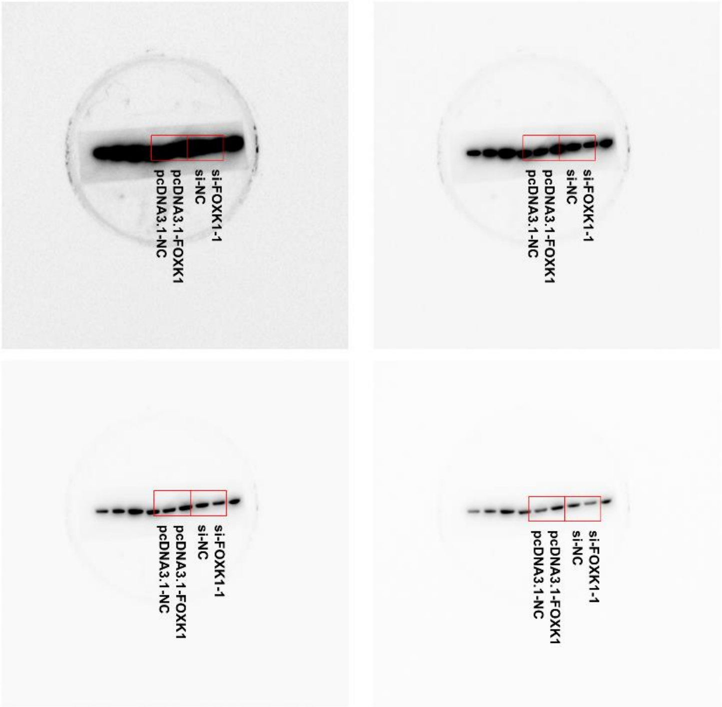

FOXK1 (protein bands of FOXK1 in KYSE170 and KYSE150 cells with different transfection treatments in Figure 5d).

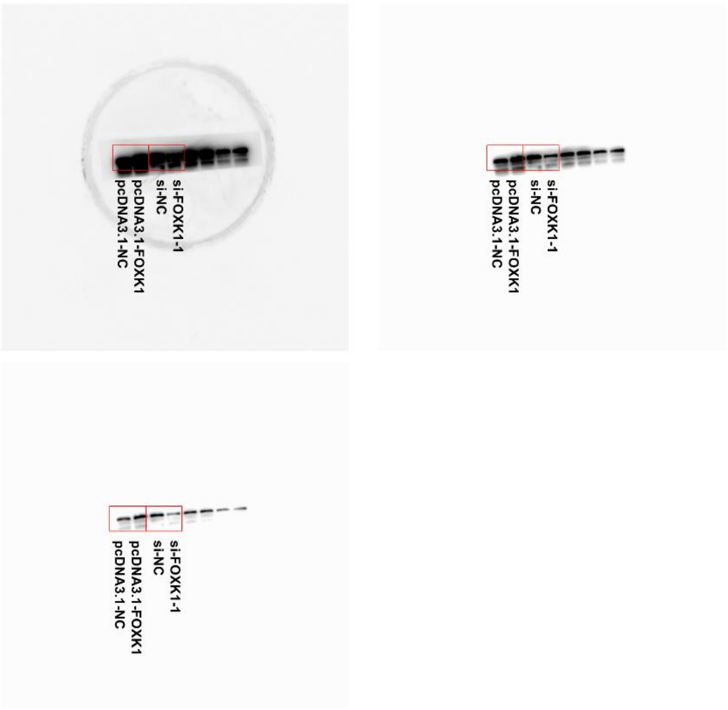

CDC25A (protein bands of CDC25A in KYSE170 and KYSE150 cells with different transfection treatments in Figure 5d).

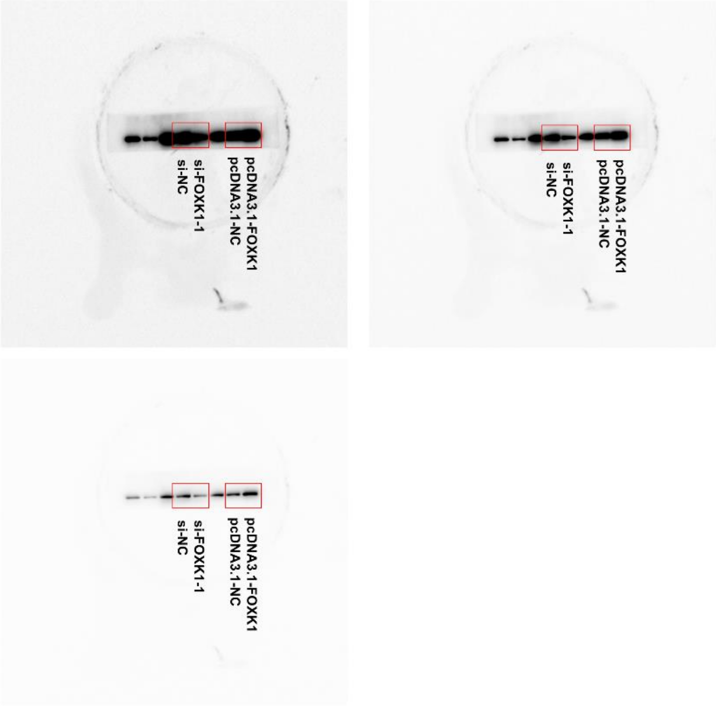

CDK4 (protein bands of CDK4 in KYSE170 and KYSE150 cells with different transfection treatments in Figure 5d).

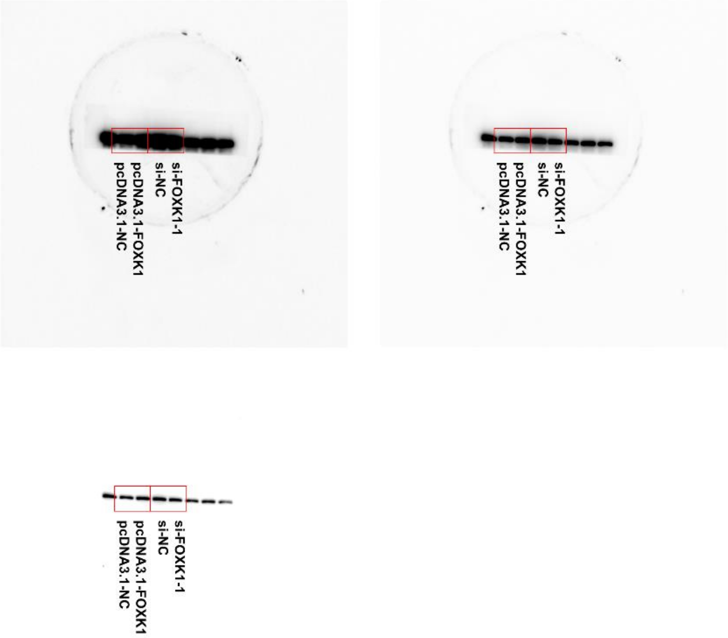

β-Actin (protein bands of β-Actin in KYSE170 and KYSE150 cells with different transfection treatments in Figure 5d).

h

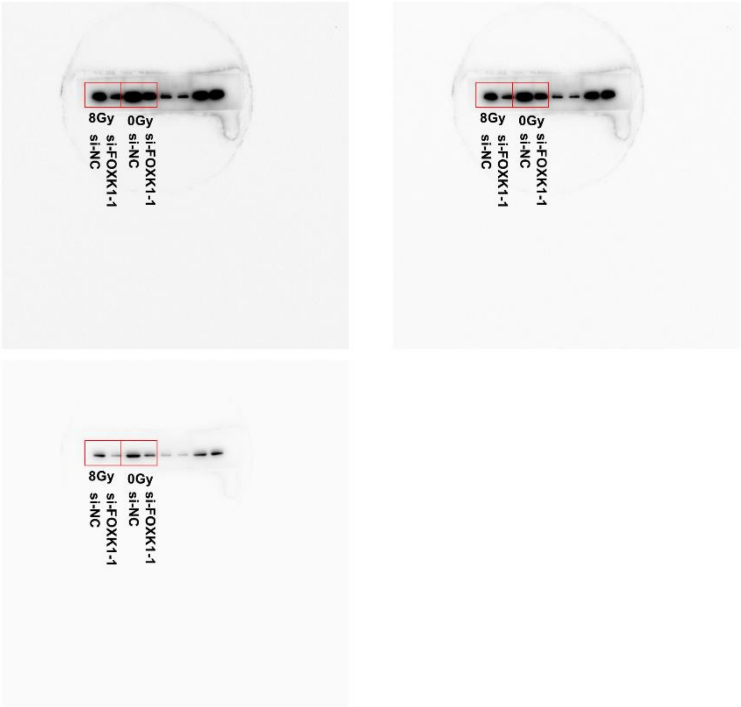

Cyclin D1 (protein bands of cyclin D1 in irradiated and non-irradiated KYSE150 cells with different transfection treatments in Supplementary Figure 2a).

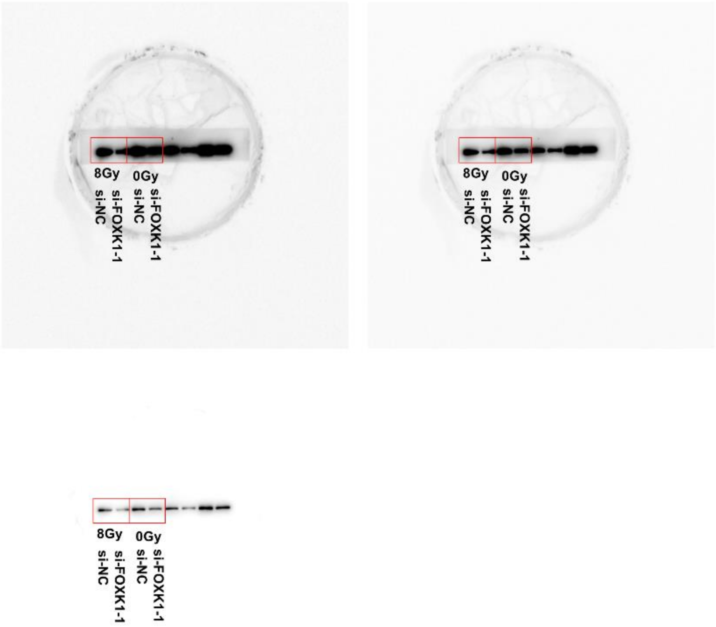

CDK4(protein bands of CDK4in irradiated and non-irradiated KYSE150 cells with different transfection treatments in Supplementary Figure 2a).

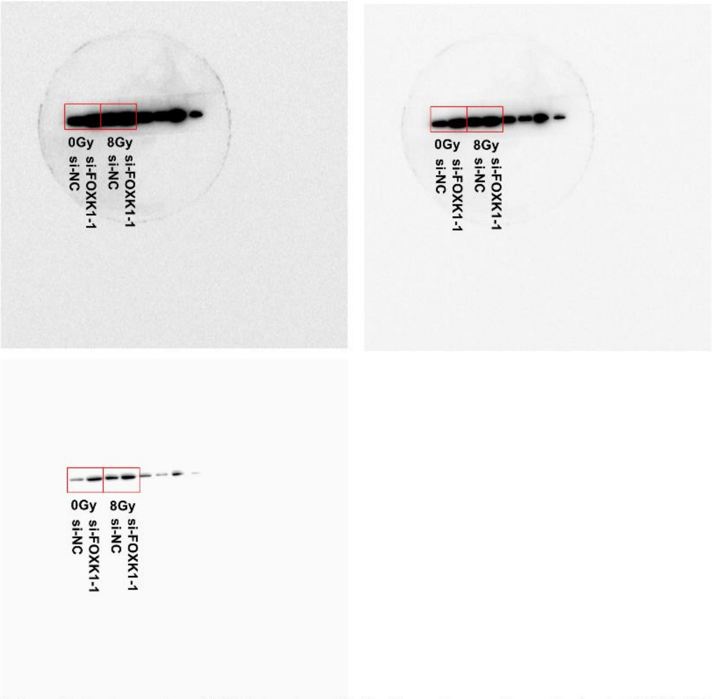

P21 (protein bands of P21 in irradiated and non-irradiated KYSE150 cells with different transfection treatments in Supplementary Figure 2a).

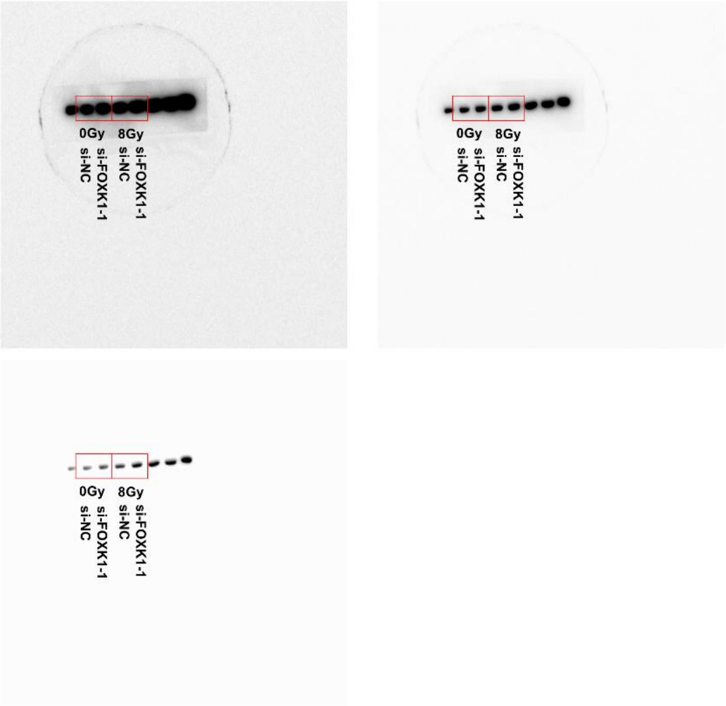

Bax (protein bands of Bax in irradiated and non-irradiated KYSE150 cells with different transfection treatments in Supplementary Figure 2a).

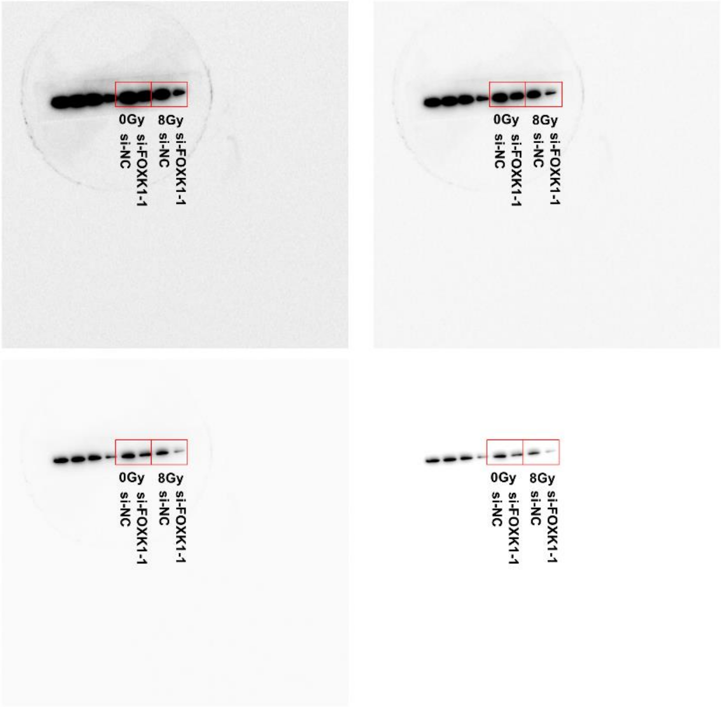

Bcl-2 (protein bands of Bcl-2 in irradiated and non-irradiated KYSE150 cells with different transfection treatments in Supplementary Figure 2a).

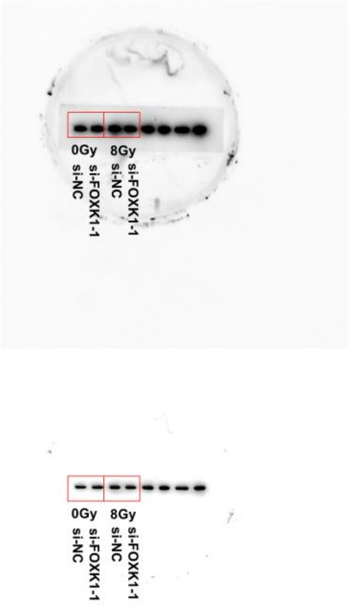

β-Actin (protein bands of β-Actin in irradiated and non-irradiated KYSE150 cells with different transfection treatments in Supplementary Figure 2a).

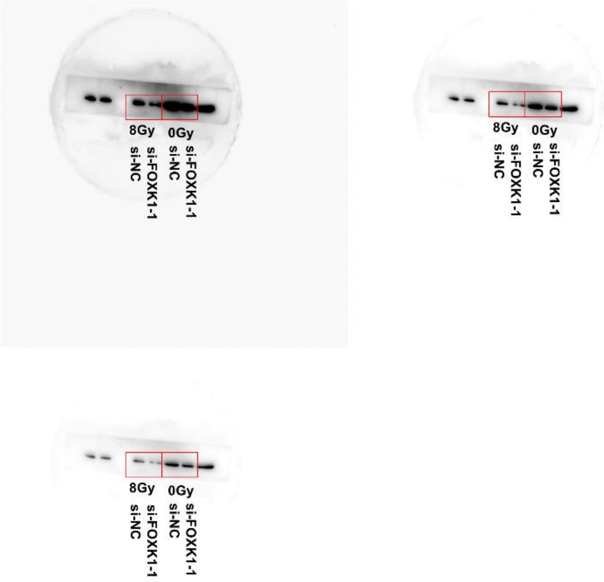

Cyclin D1 (protein bands of cyclin D1 in irradiated and non-irradiated TE1 cells with different transfection treatments in Supplementary Figure 2b).

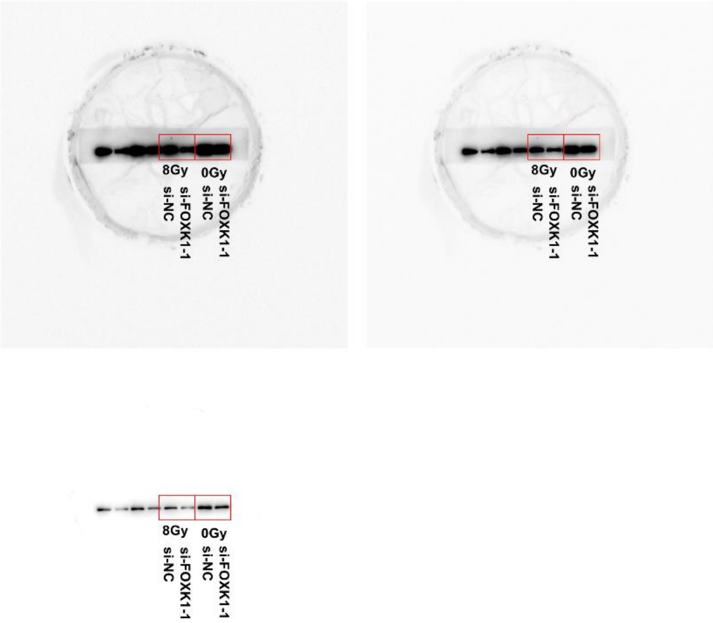

CDK4 (protein bands of CDK4 in irradiated and non-irradiated TE1 cells with different transfection treatments in Supplementary Figure 2b).

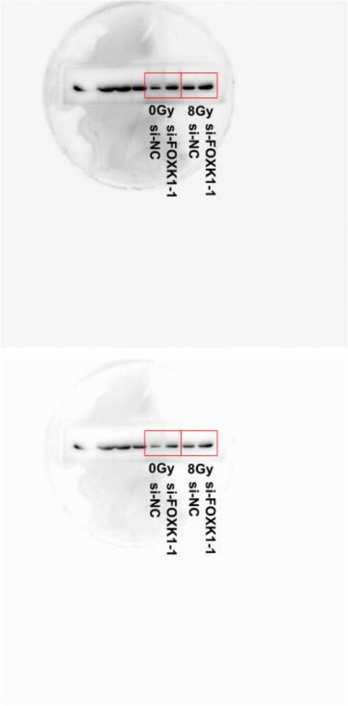

P21 (protein bands of P21 in irradiated and non-irradiated TE1 cells with different transfection treatments in Supplementary Figure 2b).

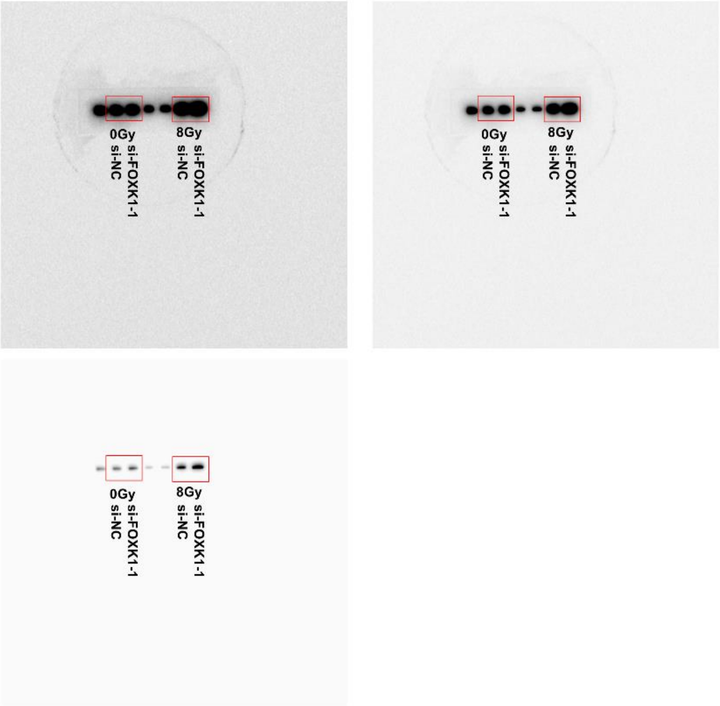

Bax (protein bands of Bax in irradiated and non-irradiated TE1 cells with different transfection treatments in Supplementary Figure 2b).

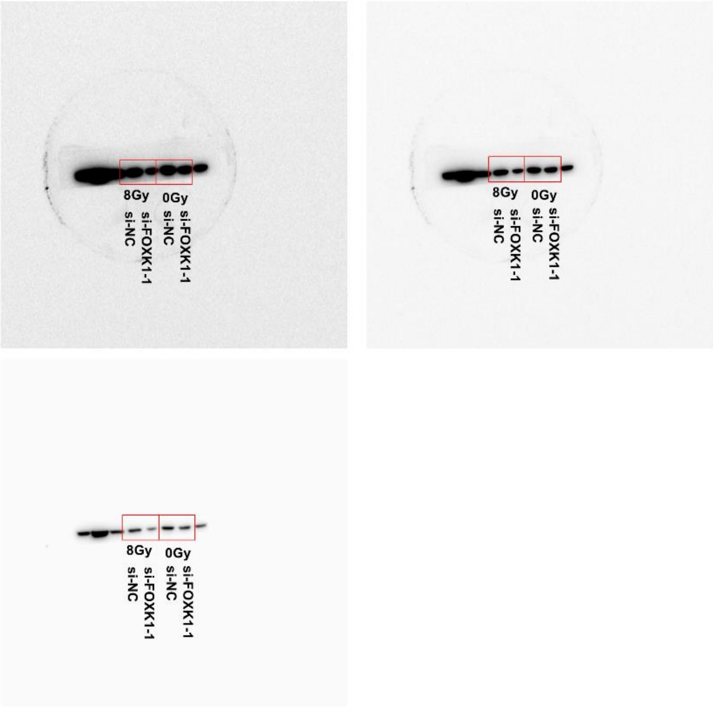

Bcl-2 (protein bands of Bcl-2 in irradiated and non-irradiated TE1 cells with different transfection treatments in Supplementary Figure 2b).

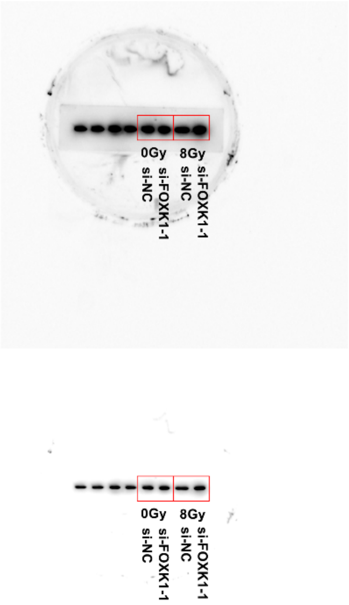

β-Actin (protein bands of β-Actin in irradiated and non-irradiated TE1 cells with different transfection treatments in Supplementary Figure 2b).

Supplementary figure 6. The original images of western blots. (**a**) The original images of the western blots in Fig. 1b. (**b**) The original images of the western blots in Fig. 3a. (**c**) The original images of the western blots in Fig. 3d. (**d**) The original images of the western blots in Fig. 3e. (**e**) The original images of the western blots in Fig. 3f. (**f**) The original images of the western blots in Fig. 3g. (**g**) The original images of the western blots in Fig. 5d. (**h**) The original images of the western blots in Supplementary Fig. 2a,2b.
